# Synergistic Neuroprotection in Parkinson’s Disease via Photobiomodulation and Liposomal Rosmarinic Acid Delivery

**DOI:** 10.1021/acsbiomaterials.5c01969

**Published:** 2026-01-26

**Authors:** Ting-Yi Su, Chen-Ya Wang, Wen-Tse Huang, Ming-Yang Chang, Ming-Hsien Chan, Ru-Shi Liu

**Affiliations:** † Department of Chemistry, 33561 National Taiwan University, Taipei 106, Taiwan; ‡ Department of Biomedical Imaging and Radiological Sciences, 34914 National Yang Ming Chiao Tung University, Taipei 112, Taiwan

**Keywords:** Parkinson’s disease, photobiomodulation, liposome, rosmarinic acid, and reactive oxygen species

## Abstract

Parkinson’s Disease (PD) is a progressive neurodegenerative disorder characterized by dopaminergic neuronal loss, oxidative stress, and mitochondrial dysfunction. Current treatment strategies are largely symptomatic and fail to halt disease progression. This research work explores a novel dual-modal therapeutic strategy combining Photobiomodulation (PBM) using near-infrared (NIR) light with nanotechnology-enhanced delivery of Rosmarinic Acid (RA) for the treatment of PD. Building upon the findings of previous works, which established the neuroprotective potential of RA, this study extends its application to PD treatment through the development of RA-loaded liposomes (RA@LP) and their integration with NIR-induced PBM. As a noninvasive modality, NIR light has demonstrated efficacy in stimulating mitochondrial activity, promoting ATP production, and reducing oxidative stress through PBM mechanisms. In parallel, RA, a potent natural antioxidant, has been encapsulated within liposomal nanocarriers to enhance its stability, bioavailability, and targeted delivery to affected neuronal tissues. The combined therapeutic platform of PBM and RA@LP is designed to eliminate endogenous and exogenous reactive oxygen species (ROS), thereby breaking the self-perpetuating cycle of oxidative stress and mitochondrial damage underlying PD pathogenesis. We highlight *in vitro* investigations that demonstrate the synergistic effects of PBM and RA@LP on neuronal cells. The results indicate that this dual approach protects mitochondrial integrity and improves cellular viability under PD-like oxidative conditions. By broadening the scope to include *in vitro* analysis, the study provides deeper mechanistic insights into the cellular responses to light-based and nanomedicine therapies. This work presents a promising, noninvasive, and multitargeted strategy for PD treatment, with potential implications for translational research. Integrating phototherapy and nanotechnology represents a significant advancement in developing effective neuroprotective interventions.

## Graphical abstract

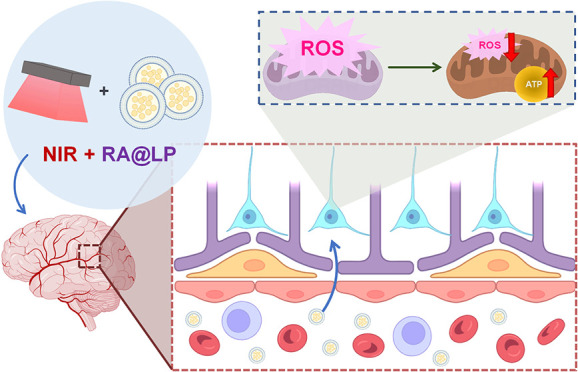

## Introduction

Parkinson’s Disease (PD) is a debilitating neurodegenerative disorder that affects millions worldwide, primarily characterized by the progressive degeneration of dopaminergic neurons in the substantia nigra.^1^ Clinically, this manifests as motor dysfunction, including tremors, rigidity, and bradykinesia, alongside nonmotor symptoms such as depression and cognitive decline.^2^ While current pharmacological treatments—like l-DOPA and dopamine agonists—offer temporary symptom relief, they fail to alter disease progression and often lead to long-term side effects.^3^ Thus, there is an urgent and unmet need for novel, disease-modifying therapies that target PD’s underlying cellular and molecular mechanisms. A growing body of evidence has highlighted oxidative stress and mitochondrial dysfunction as central drivers in the pathogenesis of PD.^4^ Reactive oxygen species (ROS) generated endogenously by mitochondrial metabolism and exogenously by neuroinflammation contribute to a self-perpetuating cycle of neuronal damage.^5^ Mitochondria, being both the source and the target of ROS, are particularly vulnerable, and their impairment exacerbates neuronal energy failure and cell death. Therefore, interrupting this vicious cycle of oxidative stress and mitochondrial dysfunction represents a promising strategy for neuroprotection in PD.^6^

In recent years, photobiomodulation (PBM)—applying low-level near-infrared (NIR) light to biological tissues—has emerged as a noninvasive modality capable of modulating mitochondrial function and promoting cellular repair.^7^ NIR light can penetrate deep into biological tissues and stimulate mitochondrial cytochrome c oxidase, enhancing ATP production and reducing oxidative stress.^8^ PBM has been shown to exert neuroprotective effects in various preclinical models of neurodegeneration, making it a compelling candidate for PD therapy.^9^ However, while PBM is promising, combining it with targeted antioxidant therapy could significantly enhance its therapeutic efficacy.^10^

Rosmarinic Acid (RA), a naturally occurring polyphenolic compound found in herbs like rosemary and perilla, possesses potent antioxidant, anti-inflammatory, and neuroprotective properties.^11^ Preclinical studies have demonstrated that RA can scavenge ROS, inhibit proinflammatory mediators, and protect neuronal cells from apoptosis. However, when administered systemically, RA’s clinical application is hindered by poor water solubility, instability in physiological conditions, and limited bioavailability.^12^ These limitations necessitate an advanced delivery system to realize RA’s full therapeutic potential in PD. To address this challenge, liposomal nanocarriers (RA@LP) are employed in this study to encapsulate and deliver RA in a stable, bioavailable form.^13^ Liposomes, composed of biocompatible phospholipid bilayers, protect RA from degradation, enhance its solubility, and enable targeted delivery to neural tissues.^14^ Moreover, the nanocarrier design allows sustained release and better cellular uptake, ensuring that therapeutic concentrations of RA are maintained within the affected brain regions.^15^ When paired with PBM, liposome-mediated delivery provides a platform for dual-action therapy, combining the benefits of light-stimulated cellular repair with targeted biochemical intervention.^16^ The novelty of this work lies in integrating PBM and RA-loaded liposomes into a synergistic therapeutic platform aimed at disrupting the core pathological mechanisms of PD. Simultaneously neutralizing both endogenous and exogenous ROS prevents mitochondrial collapse and interrupts the feedback loop of oxidative damage. This dual-modality approach not only amplifies the individual benefits of PBM and RA but also introduces a new dimension of control and precision to neurodegenerative disease treatment.^17^

This study extends its investigation by incorporating *in vitro* cellular systems, providing mechanistic insight into how PBM and RA@LP interact at the cellular level. These models allow for detailed analysis of mitochondrial dynamics, ROS scavenging, and neuronal viability, laying the groundwork for future translational studies. The integrated use of light-based therapy and nanotechnology represents a cutting-edge strategy with broad implications not only for PD but also for other oxidative stress-related neurodegenerative disorders. Our research investigates a novel dual-modal therapy combining PBM and RA@LP for treating PD (Figure 1). RA exhibits antioxidant activity primarily through its ability to scavenge free radicals, chelate metal ions, and upregulate endogenous antioxidant enzymes such as superoxide dismutase (SOD) and catalase.^18^ It interrupts oxidative stress pathways by neutralizing ROS, thus protecting cellular components like lipids, proteins, and DNA from oxidative damage.^19^ NIR PBM enhances mitochondrial function by stimulating cytochrome c oxidase (complex IV of the electron transport chain), leading to increased ATP production, improved mitochondrial membrane potential, and reduced oxidative stress.^20^ This activation supports cellular energy metabolism and provides protection against mitochondrial dysfunction under various pathological conditions (Figure S1).

**Figure 1:**
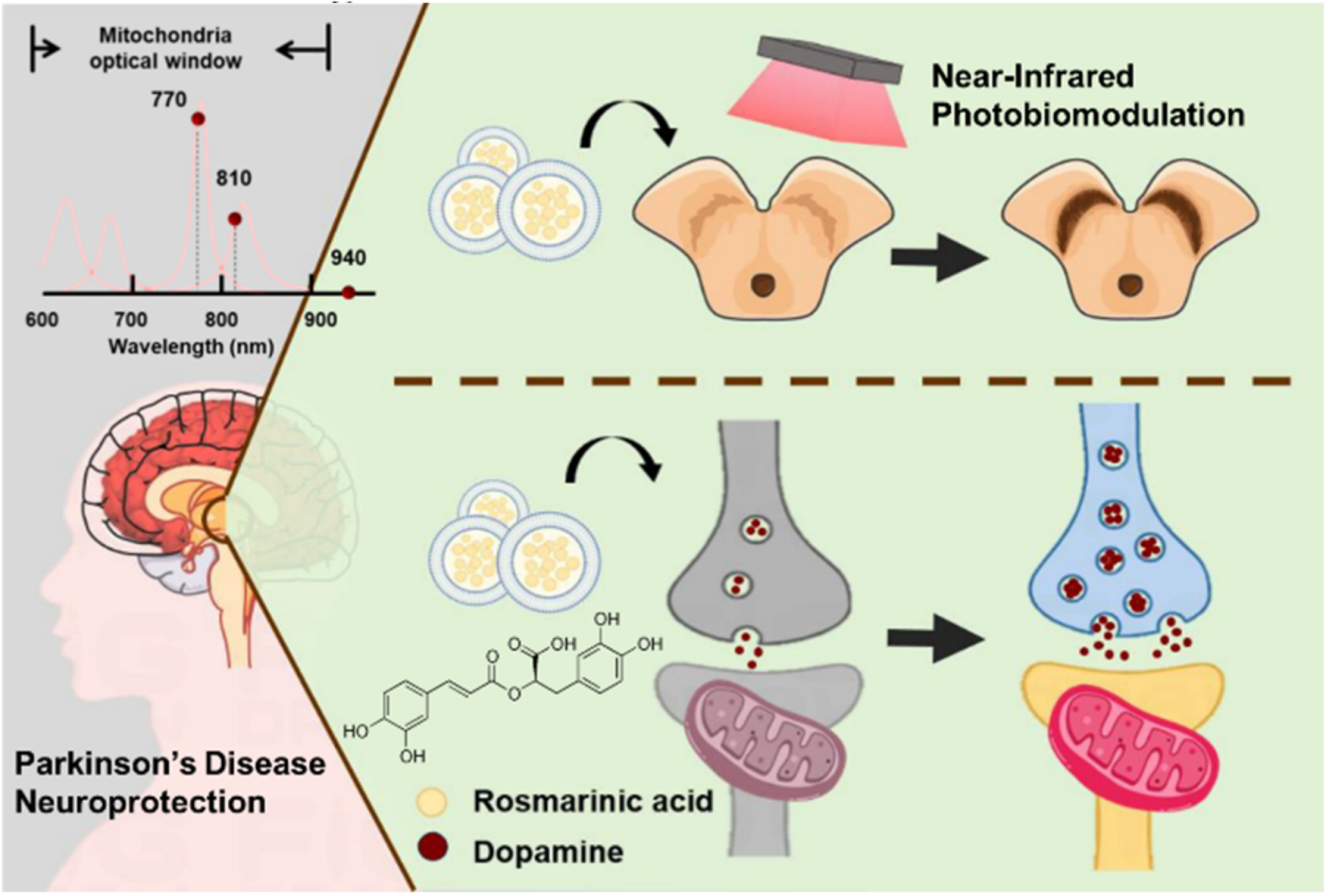
NIR PBM activates cytochrome c oxidase in mitochondria, boosting ATP production and stabilizing mitochondrial function, thereby protecting cells from oxidative damage. Meanwhile, RA@LP reduces oxidative stress by scavenging reactive oxygen species, chelating metal ions, and enhancing antioxidant enzymes.

The as-synthesized RA@LP possesses good stability and desirable sizes and shows a fine ROS cleavage capability. For *in vitro* studies, the PD model was built successfully, while the treatment combining RA@LP and 770 nm exhibited an elaborate, advanced therapeutic effect, achieving a 21% recovery rate in cell viability. Intracellular ROS levels, mitochondrial function, and SOD content analysis further confirmed the synergistic effect, with the combination therapy achieving the highest recovery of antioxidant capacity, reaching 49% of normal SOD levels with RA@LP + 770 nm treatment.

## Results and Discussion

### Morphological and Spectral Analyses of RA@LP

The synthesis of rosmarinic acid-loaded nanoliposomes (RA@LP) is similar to that of standard liposomes. Lipid components are dissolved in ethanol along with RA, and the solvent is evaporated at room temperature to form a thin lipid film. A surfactant solution is then added, and the mixture is shaken uniformly, followed by ultrasonication to form nanosized liposomes. Finally, the suspension is filtered to remove unencapsulated drug and to standardize particle size, resulting in the formation of RA@LP (Figure S2). The morphology of LP and RA@LP was examined using TEM, as shown in Figure 2a[Fig fig2],b. The preliminary particle size measurements were conducted using ImageJ software, revealing an average diameter of 126 ± 10 nm. TEM images confirmed the spherical structure of RA@LP. The size distribution and ζ-potential measurements in PBS were further tested by DLS, showing that both LP and RA@LP maintained average particle sizes between 130 and 140 nm (Figure 2c), which indicated that RA encapsulation did not significantly alter the particle size. These dimensions are favorable for biological applications. As shown in Figure 2d, ζ-potential measurements showed a change in the liposome from −21.3 to −6.3 mV after incorporation of RA. This shift suggests RA integration into the lipid bilayer and subsequent structural rearrangement. The increase in ζ-potential likely results from pH changes caused by the dissociation of RA’s carboxyl groups. RA exhibits a characteristic absorption peak at 328 nm, as shown in Figure 2e. Comparative absorption spectra of LP and RA@LP reveal RA’s characteristic absorption peak at 324 nm in the RA@LP spectrum, confirming successful RA-LP complexation. The loading concentration of RA@LP was further characterized as 46% via the calibration curve, as shown in Figure 2f. Upon excitation at 328 nm, RA exhibits a fluorescence emission peak at 432 nm, as shown in Figure 2g. The absorbance of RA at 328 nm increases proportionally with concentration, whereas its fluorescence intensity does not show a linear correlation due to concentration-dependent quenching effects. To evaluate the stability of LP and RA@LP for potential systemic circulation applications, their particle size changes were monitored over six h in deionized water and PBS.

**Figure 2:**
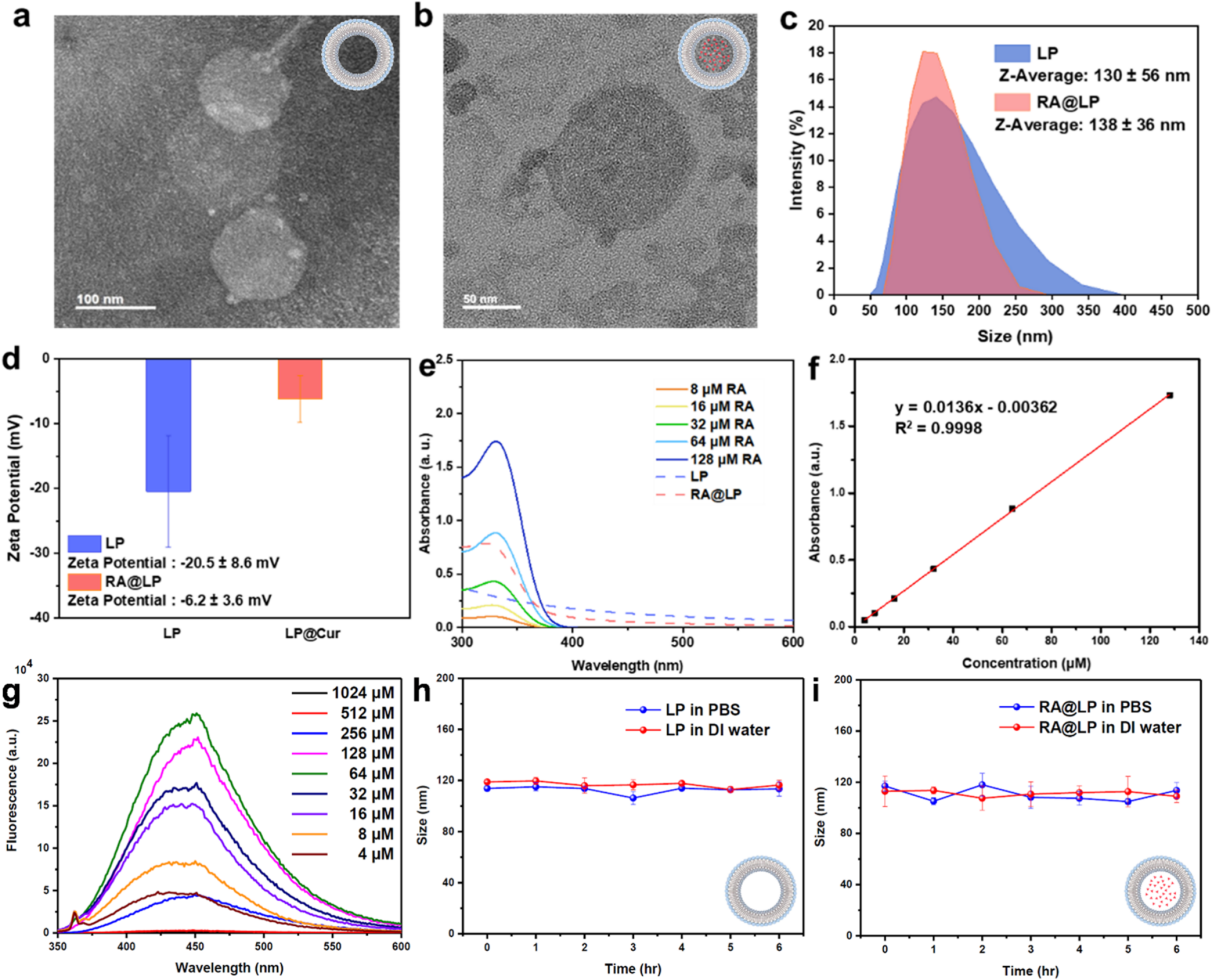
Characterization of RA@LP. TEM image of (a) LP and (b) RA@LP. (c) Size distribution, (d) ζ-potential, and (e) UV–vis absorption of LP and RA@LP. (f) Calibration curve for RA quantification. (g) The emission spectra of RA at various concentrations in ethanol were recorded using an excitation wavelength of 328 nm. Stability test results of (h) LP and (i) RA@LP in deionized water and PBS show their behavior under different aqueous environments.

As shown in Figure 2h[Fig fig2],i, both LP and RA@LP maintained consistent peak sizes within the range of 100–120 nm, with no significant shifts or aggregation observed. The size distributions and stability of LP and RA@LP in the serum solution are also analyzed. The average particle sizes of LP and RA@LP decreased slightly in serum, likely due to differences in measurement conditions (Figure S3a,b). Notably, both formulations maintained stable size distributions without significant aggregation over 96 h in fetal bovine serum (FBS), demonstrating good serum compatibility and colloidal stability, as shown in Figure S3c. These results indicate that LP and RA@LP are stable in aqueous, phosphate-buffered saline, and serum conditions, suggesting favorable pharmacokinetic properties for in vivo use.

### Hemolysis Assay

The biocompatibility of the developed formulations was initially assessed through hemolysis testing. As shown in Figure 3a, a photograph of the hemolysis test tubes revealed distinct differences between the control groups and test samples. The negative control (N) displayed no visible hemolysis, maintaining the characteristic pale yellow color of plasma, while the positive control (P) exhibited complete hemolysis with a deep red coloration indicative of extensive red blood cell lysis. Both LP and RA@LP formulations demonstrated barely color change, closely resembling the negative control, suggesting excellent hemocompatibility. Quantitative analysis of hemolysis rates (Figure 3b) corroborated these visual observations. Though the hemolysis rate showed a slightly dose-dependent increasing tendency, the LP and RA@LP groups exhibited similarly low hemolysis rates, negligible hemolysis (<10%), indicating that both LP and RA@LP possess excellent blood compatibility, making them suitable for intravenous administration without risk of hemolytic complications.

**Figure 3:**
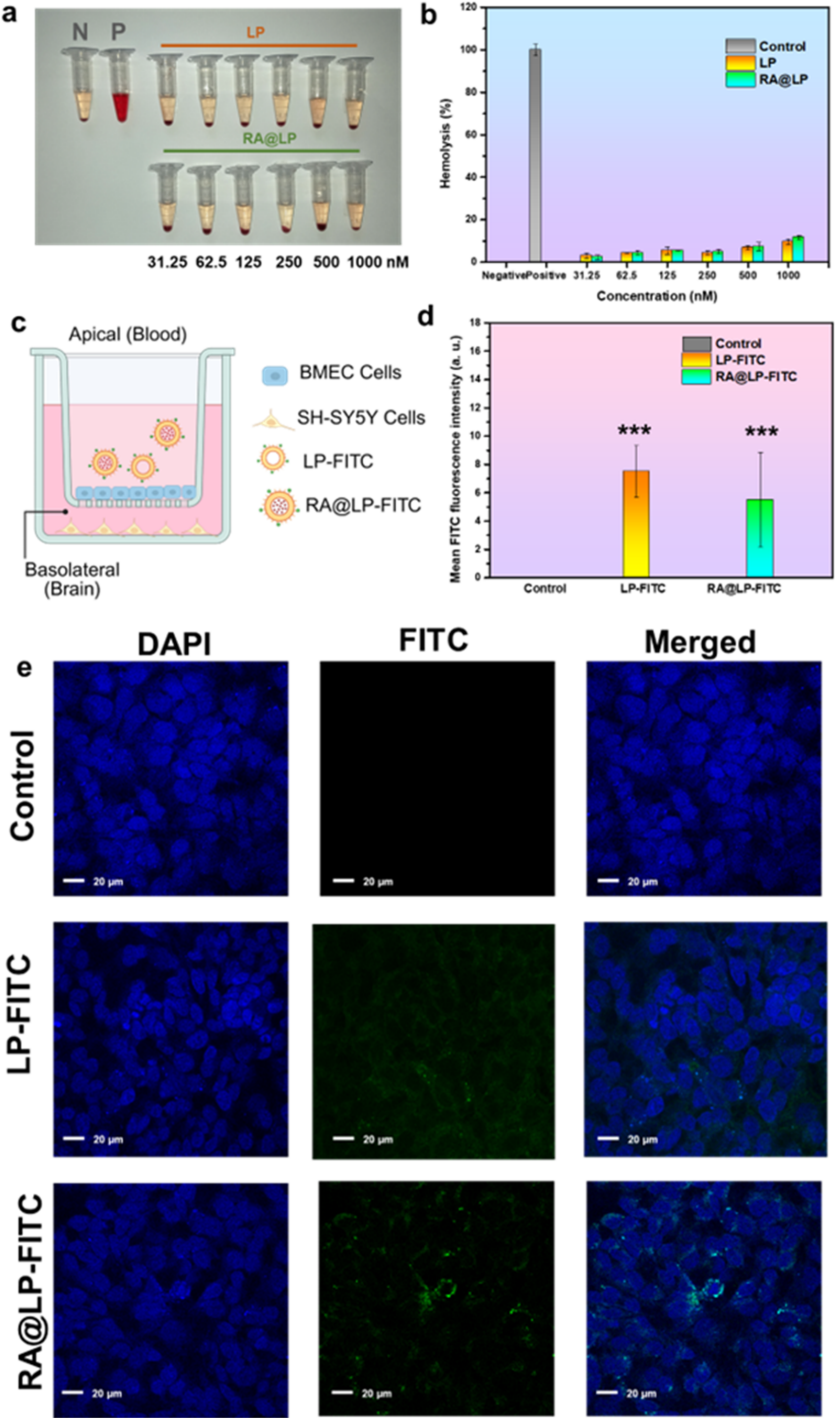
Biocompatibility assay of LP and RA@LP formulations. (a) Photograph of hemolysis test tubes and (b) hemolysis assay of LP and RA@LP. (c) The schematic diagram of the Transwell system design mimicking the BBB. The FITC-tracking BBB penetration results of (d) fluorescence intensity and (e) confocal microscopy (*p < 0.05, **p < 0.01, ***p < 0.001 compared to the control group).

#### Blood–Brain Barrier (BBB) Penetration Assay

To evaluate the brain-penetrating capability of the developed formulations, an in vitro blood–brain barrier (BBB) model was established by using a Transwell system (Figure 3c). The model consisted of a dual-chamber configuration separated by a semipermeable membrane, with the upper chamber representing the blood compartment and the lower chamber simulating the brain tissue environment, as shown in Figure S4. Brain microvascular endothelial cells were cultured on the membrane to mimic the BBB’s selective permeability characteristics.

For tracking purposes, both LP and RA@LP formulations were labeled with fluorescein isothiocyanate (FITC), allowing for monitoring of their transcellular transport. The BBB penetration efficiency was quantitatively assessed by measuring the fluorescence intensity in the brain compartment after incubation with FITC-labeled formulations. As depicted in Figure 3d, significant differences were observed among the treatment groups. The control group showed baseline fluorescence levels, while the LP-FITC and RA@LP-FITC groups demonstrated significantly enhanced fluorescence intensity (***p < 0.001). The substantial improvement in BBB penetration can be attributed to the liposomal formulation, which likely facilitates penetration across the brain endothelial barrier. Confocal microscopy provided visual confirmation of the BBB penetration results (Figure 3e). In the control group, minimal green fluorescence was observed, confirming the integrity of the BBB model and absence of nonspecific permeation. The LP-FITC group and RA@LP-FITC treatment both displayed a markedly enhanced FITC signal, suggesting successful transcellular transport and accumulation in the brain compartment. The signal was primarily localized near the cellular boundaries, suggesting a possible endocytic uptake and fine distribution within the target cells. The progressive increase in fluorescence intensity from the control to LP and RA@LP groups, as visualized in these confocal images, provides compelling evidence for the superior brain-penetrating capability of the liposomal system.

### RA-Encapsulation and Antioxidative Analysis

Subsequent measurements of the absorption spectra of RA@LP with varying concentrations of encapsulated RA are shown in Figure S5. Using the absorbance at 328 nm along with the calculated ideal absorbance values, the encapsulation efficiency and loading concentration were determined using eqs 3 and 4. As shown in Figure 4a, RA@LP with 100 μM RA achieved an encapsulation efficiency of 93%, indicating that liposomes serve as an effective nanocarrier for RA. The DPPH assay measures antioxidant activity using a lipophilic free radical.^21^ When antioxidants reduce DPPH, their color changes from deep purple to pale yellow, corresponding to a decreased absorption at 517 nm. While LP showed no significant radical scavenging activity, RA and RA@LP demonstrated over 90% reduction in ROS levels compared to the control group (set at 100%), as shown in Figure 4b. These results indicate that RA possesses potent antioxidant properties and that its encapsulation in LP does not compromise its radical scavenging capability. Furthermore, the stability of RA was inspected through a DPPH assay, as shown in Figure 4c. Free RA rapidly degraded in PBS, retaining only 20% of its antioxidant activity after 2 h. In contrast, RA@LP maintained 62% of its antioxidant capacity after 3 h in PBS, demonstrating that liposomal encapsulation significantly enhanced RA’s stability.

**Figure 4:**
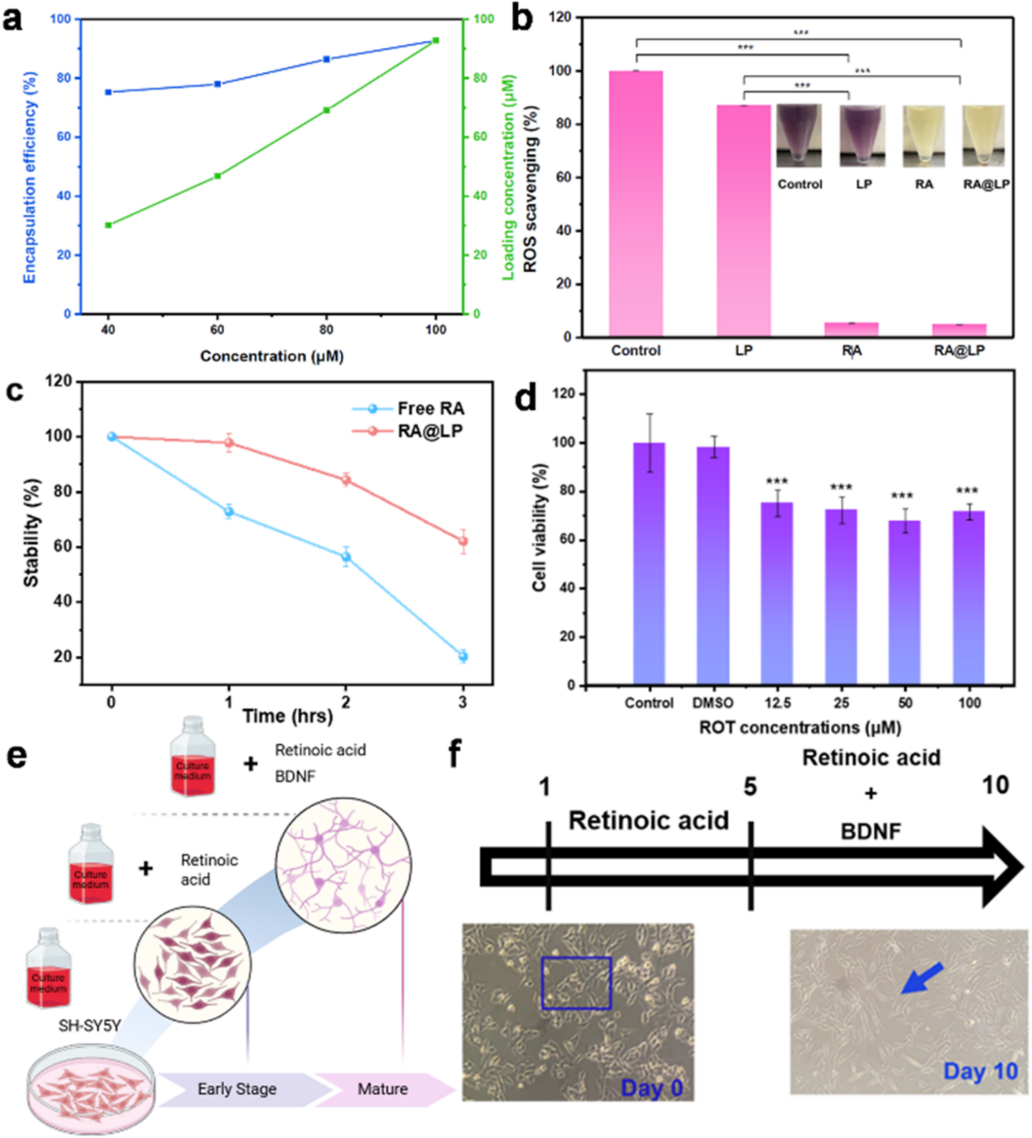
(a) Line graph illustrating the encapsulation efficiency (EE) and loading concentration of RA@LP at various RA concentrations shows the relationship between the input RA concentration and the resulting encapsulation performance. (b) ROS remaining ratio (inset: color change diagram of DPPH solution after reaction with the drug). (c) The stability assay was based on DPPH scavenging (*p < 0.05, **p < 0.01, ***p < 0.001 compared to the control group). (d) The rotenone-induced PD models. (e) The differentiation scheme of SH-SY5Y cells and (f) observing the cell morphology under the optical microscope.

### Cell Differentiation and Rotenone (ROT)-Induced Model

SH-SY5Y cells were differentiated according to the protocol.^22^ Retinoic acid treatment increased neurite outgrowth and elongated the cell bodies in the first differentiation phase. The second phase, involving brain-derived neurotrophic factor (BDNF), promoted neuronal growth and synaptic formation (Figure 4e).^23^ As shown in Figure 4f, differentiated cells exhibited a triangular and flattened morphology. The cells displayed elongated morphology with fine, extended neurites, indicating successful differentiation suitable for subsequent Parkinson’s disease (PD) model experiments.^24^ To establish the PD cell model, ROT was used as an inducer, and a cell viability test was performed to optimize the induction concentration.^25^ Cell viability measurements following different ROT concentrations are shown in Figure 4d. ROT treatment significantly reduced cell viability to below 80% compared with the control group. The 50 μM ROT treatment resulted in 68% cell viability, which aligned with the optimized concentration in the literature and was therefore selected as the optimal concentration for establishing the PD cell model.

### Cell Viability under Treatment

To induce SH-SY5Y cells to differentiate into a suitable neuronal phenotype for PD research, this study employed a stepwise regulation of the culture medium for differentiation (Figure S6).^26^ After the differentiation process, we evaluated the effect of nanoliposomes combined with RA on PD model cells. The differentiated cells were seeded into 96-well plates and incubated in a cell culture incubator at 37 °C with 5% carbon dioxide for a period of time. Subsequently, a neurotoxin solution was added to induce PD-like conditions in the cells, followed by another incubation period. After induction, the cells were treated with nanoliposomes containing RA and exposed to light panels at wavelengths of 770, 810, and 940 nm for a short duration. The cells were then returned to the incubator for further cultivation (Figure S7). The cell viability tests were conducted to assess the biosafety of RA and RA@LP. As shown in Figure 5a, the cell viability remained above 90% across all tested concentrations of both RA and RA@LP, demonstrating good biocompatibility with SH-SY5Y cells. In the PD model, SH-SY5Y cells were first treated with 50 μM ROT to induce Parkinsonian conditions, followed by RA or RA@LP treatment.^27^ As shown in Figure 5b, ROT induction significantly reduced cell viability to 68% compared to controls. After 24-h treatment. Treatment groups showed significant improvement compared to the PD model group (with significance marked with #), with a maximum recovery rate of 10% achieved at 80 μM RA. For RA@LP treatment, all concentrations showed significant improvement in cell viability, with an optimal recovery rate of 20% observed at 40 μM RA@LP. Notably, at 40 μM, RA@LP showed 2.8 times better recovery than 80 μM RA. These results indicate that liposomal encapsulation enhances RA’s therapeutic efficacy, achieving better outcomes at lower doses. The improved effectiveness likely results from enhanced cellular uptake, which allows RA to neutralize ROT-induced ROS more effectively and prevent cell apoptosis. Based on these findings, 40 μM RA@LP was selected as the optimal concentration for subsequent studies. A biocompatibility study was conducted to evaluate the potential harmful effects of NIR exposure on cells. As shown in Figure 5c, SH-SY5Y cells were exposed to NIR at wavelengths 770, 810, and 940 nm for 15 min, followed by a 24 h incubation period. Cell survival rates showed no significant difference compared to the control group. Interestingly, the groups treated with 770 and 940 nm wavelengths showed survival rates slightly exceeding 100%. This phenomenon is likely due to the mitochondrial absorption of infrared light, which enhances electron transport across mitochondrial membranes and increases ATP production and cellular proliferation. Overall results indicate that NIR exposure has no cytotoxic effects on SH-SY5Y cells. The therapeutic effects of PBM utilized 770, 810, and 940 nm NIR, as shown in Figure 5d. Among the PBM treatments of the three wavelengths, the 770 nm treatment showed the best recovery rate at 12%, while the 810 and 940 nm treatments showed recovery rates of 11 and 10%, respectively. These results demonstrate that PBM has therapeutic potential. RA reduces cell death rates by lowering the levels of ROS through its antioxidant properties. NIR increases cell survival rates by promoting electron transport chain activity in mitochondria. This study combined these two therapeutic approaches to achieve better treatment outcomes for PD. We investigated whether the combined treatment using RA@LP drug delivery and NIR PBM had superior therapeutic effects. As shown in Figure 5e, the combined treatment groups using both drug therapy and PBM, the complexation of 40 μM RA@LP with 770 nm light exposure, demonstrated the most effective therapeutic outcome, achieving a 21% recovery rate. The combinations using 810 and 940 nm light exposure showed recovery rates of 14 and 13%, respectively. These results indicate that while drug treatment alone and PBM alone showed similar recovery rates, combining these two therapeutic approaches produced a multiplicative effect, reaching a 21% recovery rate. This represents double the recovery rate compared to drug treatment alone and 1.8 folds of the recovery rate of PBM alone. The highest recovery rate was observed with the 770 nm wavelength treatment group. This superior performance is likely due to this wavelength being most compatible with mitochondrial absorption spectra, thus producing optimal PBM results. In Figure 5f, the synergistic effects of NIR and RA@LP were assessed through evaluation of the Bliss synergy score. The Bliss synergy score is a quantitative metric that evaluates the interactions between multiple treatments, where positive values indicate synergistic effects and negative values indicate antagonistic effects between two therapeutic modalities. Following the analysis of cell viability, PBM at 770, 810, and 940 nm wavelengths combined with RA@LP demonstrated Bliss synergy scores of −0.12, −0.32, and −0.31, respectively, revealing mild antagonistic effects. Among these combinations, the 770 nm and RA@LP pairing exhibited the minimal antagonism. Despite these antagonistic trends, the combination of NIR and RA@LP treatment demonstrated superior therapeutic efficacy compared to either monotherapy alone. While cell viability serves as a primary indicator of proliferative synergy for assessing therapeutic effectiveness, this metric alone is insufficient for evaluating the efficacy of cellular treatment in PD disease models. Cell viability does not account for critical functional parameters, such as neuronal activity and mitochondrial function, which are essential for restoring motor control and neural function in Parkinson’s disease pathology. Therefore, we further investigated the ATP generation profiles to provide a more mechanistic and comprehensive evaluation of treatment outcomes, with a particular focus on mitochondrial performance as a key indicator of neuroprotection and restoration of cellular function.

**Figure 5:**
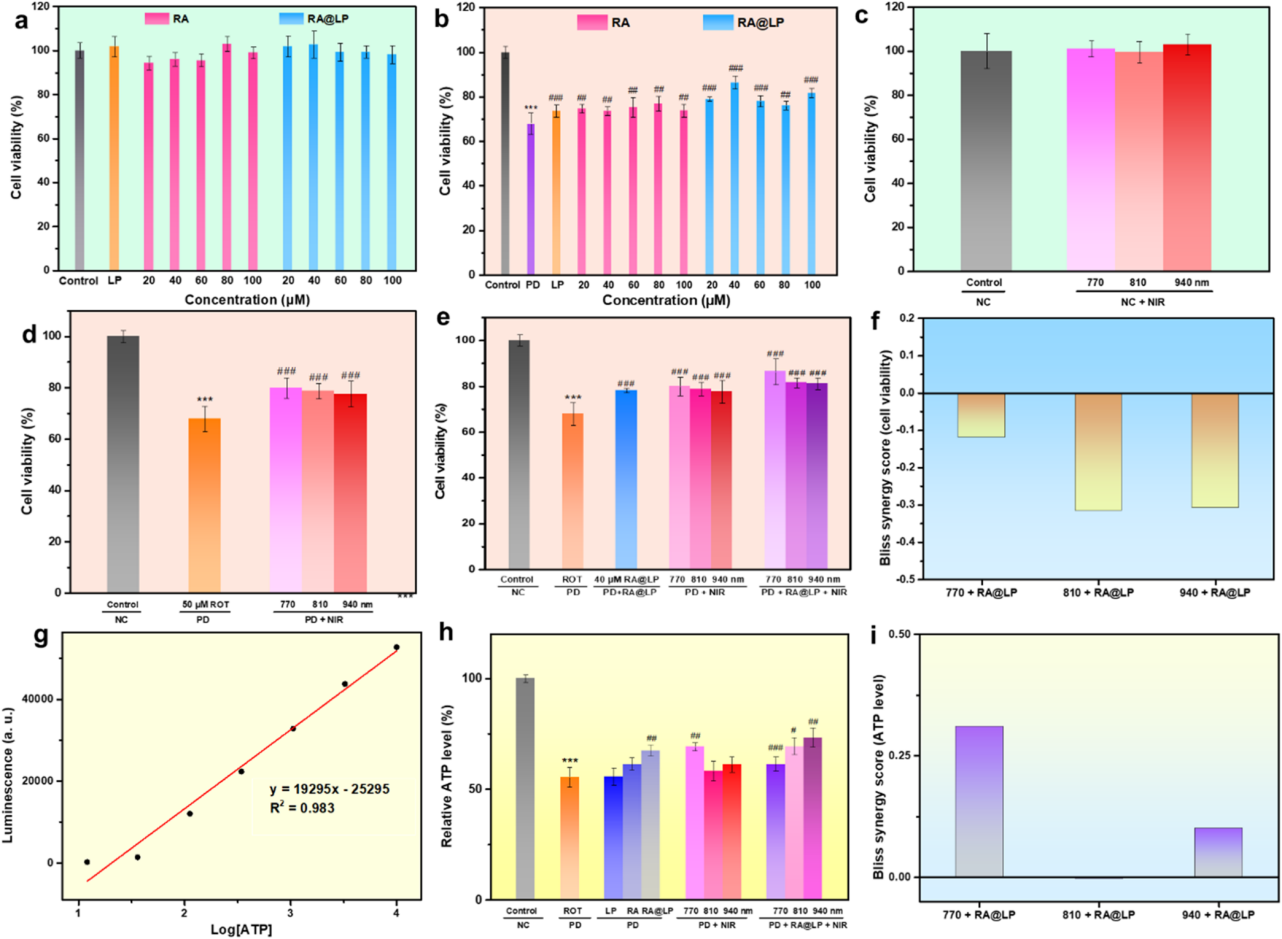
Cell viability assays of RA and RA@LP treated (a) normal SH-SY5Y cells and (b) ROT-induced PD cell model. Cell viability assays of NIR treated (c) normal SH-SY5Y cells and (d) PD model, where normal cell is denoted as NC. (e) Cell viability assays of the PD model with the RA@LP and PBM. (f) Bliss synergy score of NIR and RA@LP. (g) Scatter plot and regression curve of luminescence versus log­[ATP] for the ATP standard solution. (h) ATP level assays of the PD model with the RA@LP and PBM. (i) Bliss synergy score of NIR and RA@LP. (The significance is marked as *p < 0.05, **p < 0.01, and ***p < 0.001 compared to the control group. The significance is marked as #*p* < 0.05, ##*p* < 0.01, and ###*p* < 0.001 compared to the ROT group).

### ATP Level Assessment

ATP levels and mitochondrial activity are inseparably linked; as key indicators for evaluating cellular therapeutics in PD, the intracellular ATP levels were quantified by analyzing ATP standards. As shown in Figure 5g, the standard curve for ATP concentration was established with the equation *y* = 19295*x* – 25295 (where *A* = 19295 and *B* = −25295), and was subsequently used to quantify ATP concentrations in experimental samples. The ATP levels of treated SH-SY5Y cells are shown in Figure 5h. ROT induction significantly reduced ATP production to 55% of control levels after 24 h treatment, indicating severe mitochondrial dysfunction. Various treatment regimens substantially restored ATP levels compared to the PD model group. Single-agent treatments showed variable efficacy. RA@LP and PBM of 770 nm demonstrated superior performance with ATP recovery rates of 67 and 69%, respectively, representing increases of 12 and 14% above the ROT-induced model. In contrast, 810 and 940 showed more modest improvements, recovering to 58 and 61%, respectively. Combination treatments exhibited markedly enhanced efficacy. Most notably, RA@LP + 770 achieved exceptional mitochondrial recovery with ATP restoration to 84% of control levels, representing a striking 29% increase above the ROT-induced model and exceeding that of single-agent treatments by approximately 15%. RA@LP + 810 also showed robust recovery at 73%, while 940 achieved 69%. The data clearly demonstrate that the combination of RA@LP and 770 exhibited the most potent mitochondrial recovery effect, substantially outperforming all other treatment regimens tested. This synergistic effect suggests that the combined approach may provide an optimal strategy for restoring mitochondrial function and ATP production in PD models.

In Figure 5i, the Bliss synergy analysis further supports the existence of wavelength-dependent cooperative effects between PBM and RA@LP. The positive synergy scores observed for RA@LP + 770 nm (0.31) and RA@LP + 940 nm (0.10) indicate that the bioenergetic effects of PBM treatment amplify the antioxidant benefits. This synergy likely arises from the superior efficacy of RA@LP + 770, which is likely due to its complementary mechanisms of action that target multiple aspects of mitochondrial dysfunction. RA may promote antioxidant defense gene expression, including the upregulation of superoxide dismutase (SOD), thereby reducing the accumulation of reactive oxygen species (ROS). NIR, functioning as a mitochondria-boosting agent, likely improves electron transport chain efficiency and stabilizes the mitochondrial membrane potential. The combination of these two agents addresses ROT-induced mitochondrial damage through synergistic pathways: while RA attacks the antioxidant defense level, 770 directly enhances respiratory chain function and ATP synthase activity. Furthermore, the liposomal formulation enhances RA bioavailability and facilitates targeted delivery to damaged mitochondria, amplifying its restorative effects.

### ATP Synthase Expression Analysis

To elucidate the mechanisms underlying alterations in ATP levels, the protein expression of ATP synthase was analyzed using Western blot, as shown in Figure 6a,b, with the full gels shown in Figure S8. PD induction critically diminished ATP synthase expression to 18% of control levels, suggesting a high correlation between PD and ATP synthase deficiency. The expression trends showed a distinctly different tendency from that of the ATP level. Single-agent treatments demonstrated variable efficacy, with RA@LP and 770 nm PBM both showing similar performance with expression only around 25% each. In contrast, 810 and 940 nm treatments demonstrated greater improvements, increasing ATP synthase levels to 145 and 54% of control, respectively. In the combination treatments, RA@LP combined with 810 nm achieved an exceptional ATP synthase level of 168% of the control levels. RA@LP, combined with 770 and 940 nm, both demonstrated recovery at 124% of control levels. In Figure 6i, Bliss synergy analysis based on ATP synthase expression further substantiates the existence of wavelength-dependent cooperative effects between PBM and RA@LP. The positive synergy scores observed for RA@LP + 770 nm (6.61), RA@LP + 810 nm (0.19), and RA@LP + 940 nm (1.63) collectively indicate that the PBM treatment significantly enhances the ATP synthase expression conferred by RA@LP. The discordance between ATP synthase expression patterns and trends in ATP levels suggests that additional factors beyond ATP synthase expression may influence cellular ATP production. These findings warrant further investigation to identify alternative antioxidant-related mechanisms that contribute to ATP restoration under various treatment conditions.

**Figure 6:**
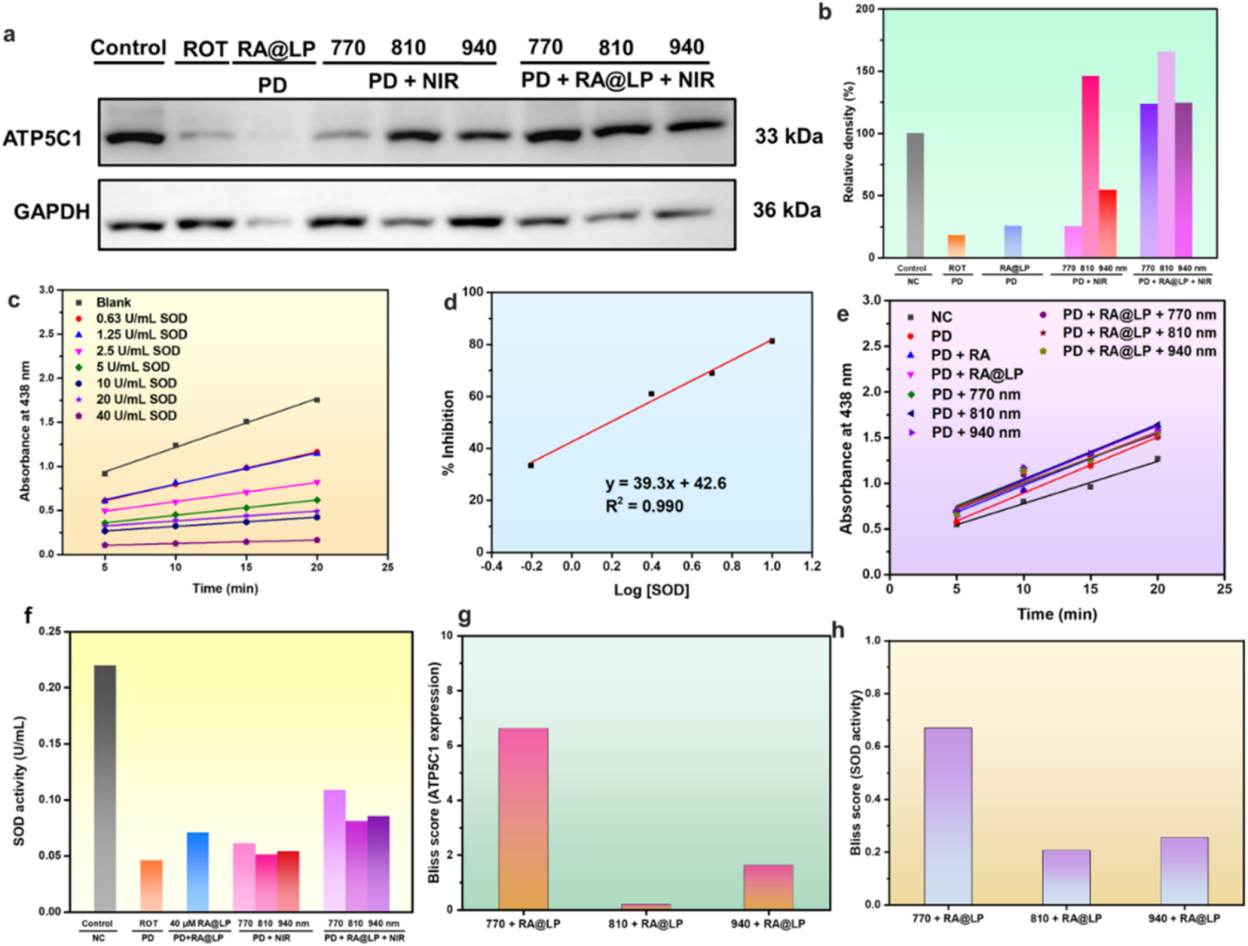
(a) Western blot image and (b) quantification histogram of ATP synthase expression. Superoxide dismutase (SOD) activity analysis with (c) scatter plot and regression curve of absorbance values versus time for the SOD standard solution, (d) scatter plot and regression curve of % inhibition versus the logarithm of concentration for the SOD standard solution, (e) scatter plot and regression curve of absorbance values versus time for samples under different treatment methods, and (f) bar graph of SOD concentrations and their relative proportions across different treatment groups. Bliss synergy score of (g) ATP5C1 expression and (h) SOD activity.

### Superoxide Dismutase (SOD) Activity Analysis

To further confirm the pathway in which PBM and RA@LP take effect, this study evaluated the antioxidant capacity of the treatment by measuring the levels of superoxide dismutase (SOD) in the cells. First, the absorbance values at 438 nm over time were recorded using SOD standards at various concentrations, as shown in Figure 6c. The regression equation of the SOD standard curve and the corresponding % inhibition values are presented in Table S1. A plot of % inhibition against the logarithm of SOD activity (log [SOD]) is shown in Figure 6d. The final standard curve equation for SOD concentration was determined to be *y* = 39.3*x* + 42.6 (*y* = *Ax* + *B*, where *A* = 39.3 and *B* = 42.6), which was used to calculate the SOD activity of experimental samples. According to the results in Table S2, higher SOD concentrations were associated with increased % inhibition of superoxide reactions, indicating greater antioxidant capacity. The regression curves of absorbance versus time and the % inhibition values for cell samples under different treatment methods are shown in Figure 6e. Using the standard curve equation *y* = 39.3*x* + 42.6 (*y* = *Ax* + *B*, with *A* = 39.3 and *B* = 42.6), the SOD concentrations of each sample group were calculated, as presented in Figure 6f. We investigate the antioxidant capacities of antioxidant drug treatment (PD + RA@LP), PBM therapy (PD + NIR), and combined antioxidant drug and PBM treatment (PD + RA@LP + NIR) by measuring the SOD content. The control group (NC) was defined with SOD activity units at 100%. After induction of Parkinson’s disease using 50 μM rotenone, the SOD level in the PD group dropped to 21% of the NC group. Treatment with PD + RA@LP increased the SOD concentration from 21 to 32%, restoring 11% of antioxidant capacity with 40 μM RA@LP. In the PD + NIR treatment groups, exposure to 770, 810, and 940 nm light raised SOD levels to 27, 24, and 23%, respectively. Among these, 770 nm PBM showed the best antioxidant recovery effect, with a 7% increase. In the combined treatment groups (PD + RA@LP + NIR), combining 40 μM RA@LP with 770, 810, and 940 nm light therapy increased SOD levels to 49, 37, and 39%, respectively. The combination of 40 μM RA@LP and 770 nm light yielded the highest antioxidant recovery rate of 28%. The superior antioxidant effect observed with 770 nm irradiation correlates with mitochondrial absorption wavelengths. These results suggest a synergistic effect between drug treatment and NIR PBM, resulting in the combined RA@LP and 770 nm light treatment group exhibiting the highest antioxidant recovery capacity. In Figure 6h, Bliss synergy analysis based on SOD activity further substantiates the existence of wavelength-dependent cooperative effects between PBM and RA@LP. The positive synergy scores observed for RA@LP + 770 nm (0.67), RA@LP + 810 nm (0.21), and RA@LP + 940 nm (0.25) collectively indicate that the PBM treatment significantly enhances the antioxidant benefits conferred by RA@LP. The cotreatment of PBM and RA@LP significantly enhanced ATP production and SOD activity, indicating synergistic effects at the level of antioxidant defense. In contrast, cell viability did not exhibit a comparable synergistic trend; however, under all treatment conditions, including PBM, RA@LP, and their combination, cell viability was consistently increased relative to that of the PD model, indicating that none of the treatments induced cytotoxic effects. The cell viability is a cumulative, relatively coarse end point that integrates multiple cellular processes, including metabolic activity, redox balance, cell cycle regulation, and stress responses. Therefore, improvements in functional and metabolic parameters may not be immediately reflected in the short-term viability assays. Notably, among the tested wavelengths, 770 nm exhibited the strongest intensifying effects, whereas 810 nm showed the weakest synergistic enhancement. This difference may help explain why the RA@LP + 810 nm group displayed a relatively high ATP synthase expression despite a comparatively lower ATP level. Cellular ATP production is governed by multiple coordinated and interdependent mitochondrial processes and is not solely determined by ATP synthase abundance. By contrast, the 770 nm PBM appears to regulate mitochondrial redox balance more effectively, as reflected by enhanced antioxidant capacity, which in turn preserves electron transport efficiency and supports ATP generation even with lower ATP synthase expression. These observations indicate that mitochondrial redox homeostasis, rather than ATP synthase expression alone, plays a critical role in determining cellular ATP levels under different PBM conditions. The superior synergistic effect achieved with RA@LP + 770 nm (0.67) can be attributed to the optimal tissue penetration and photonic energy absorption of this wavelength by cytochrome c oxidase (Complex IV) in the mitochondrial electron transport chain. PBM at 770 nm facilitates enhanced electron transfer efficiency, culminating in increased ATP generation and restoration of cellular bioenergetic capacity. Critically, the elevated ATP levels provide substantial energy support for the upregulation and synthesis of superoxide dismutase (SOD), enabling the maintenance of heightened antioxidant enzymatic activity. Furthermore, PBM-induced improvement in mitochondrial function directly reduces excessive reactive oxygen species (ROS) generation, thereby alleviating oxidative-stress-induced inhibition of SOD enzyme activity and expression.

### Analysis of Intracellular ROS Levels and Mitochondrial Function

To evaluate the relevance of intracellular ROS content in PD treatment, cells were stained with DCFH-DA dye.^28^ The results are acquired via a microplate reader and imaged using confocal microscopy to evaluate the ROS clearance efficacy, as shown in Figure 7a,d. DCFH-DA is a ROS detection dye that oxidizes to DCF, producing green fluorescence when reacting with ROS. DAPI staining was used simultaneously to identify the locations of cell nuclei by binding to cellular DNA, enabling the assessment of green fluorescence intensity in these regions to quantify ROS content. Confocal microscopy imaging results with DCFH-DA staining revealed that the PD group exhibited the most prominent green DCF fluorescence signals within cells. Following treatment with RA or RA@LP, the DCF signal intensity decreased, demonstrating the drugs’ capability to eliminate ROS. Groups treated with 770 nm wavelength therapy alone or with RA@LP displayed weaker DCF fluorescence signals. These results indicate that PBM therapy effectively reduces cellular ROS content, and the combination of RA@LP with 770 nm treatment shows effectiveness in reducing ROS levels. The flow cytometry results are presented in Figure S9a. The control group exhibited DCF fluorescence signals primarily between 10 and 10^2^, which likely represent basal ROS levels in normal cells. Both the Parkinson’s disease group (PD) and treated groups showed two distinct DCF fluorescence peaks: one between 10 and 10^2^ and another between 10^2^ and 10^3^. Comparing the PD and control groups suggests that the lower peak corresponds to normal ROS production, while the higher peak indicates cells with elevated ROS levels, reflecting greater oxidative stress. Overlay comparisons of different treatment groups, as shown in Figure S9b, reveal that after treatment, the number of cells within the 10–10^2^ fluorescence peak increased, indicating a restoration of ROS levels toward normal. This increase was most notable in the drug treatment group (PD + RA@LP) and the combined RA@LP plus 770 nm NIR therapy group (PD + RA@LP + 770 nm). Conversely, the number of cells in the higher fluorescence peak (10^2^–10^3^), representing cells under greater oxidative stress, decreased most significantly in the combined treatment group. These results indicate that the combined RA@LP and 770 nm NIR therapy effectively reduces the proportion of cells experiencing high oxidative stress, demonstrating potent antioxidant activity capable of eliminating excess ROS. Using a DCF fluorescence signal of 1 × 10^2^ as the dividing line, the proportions of cells below and above this threshold were calculated, as shown in Figure S10. The data indicate that in the control group, the majority of cells fall within the 10 to 10^2^ signal range, representing normal cells.

**Figure 7:**
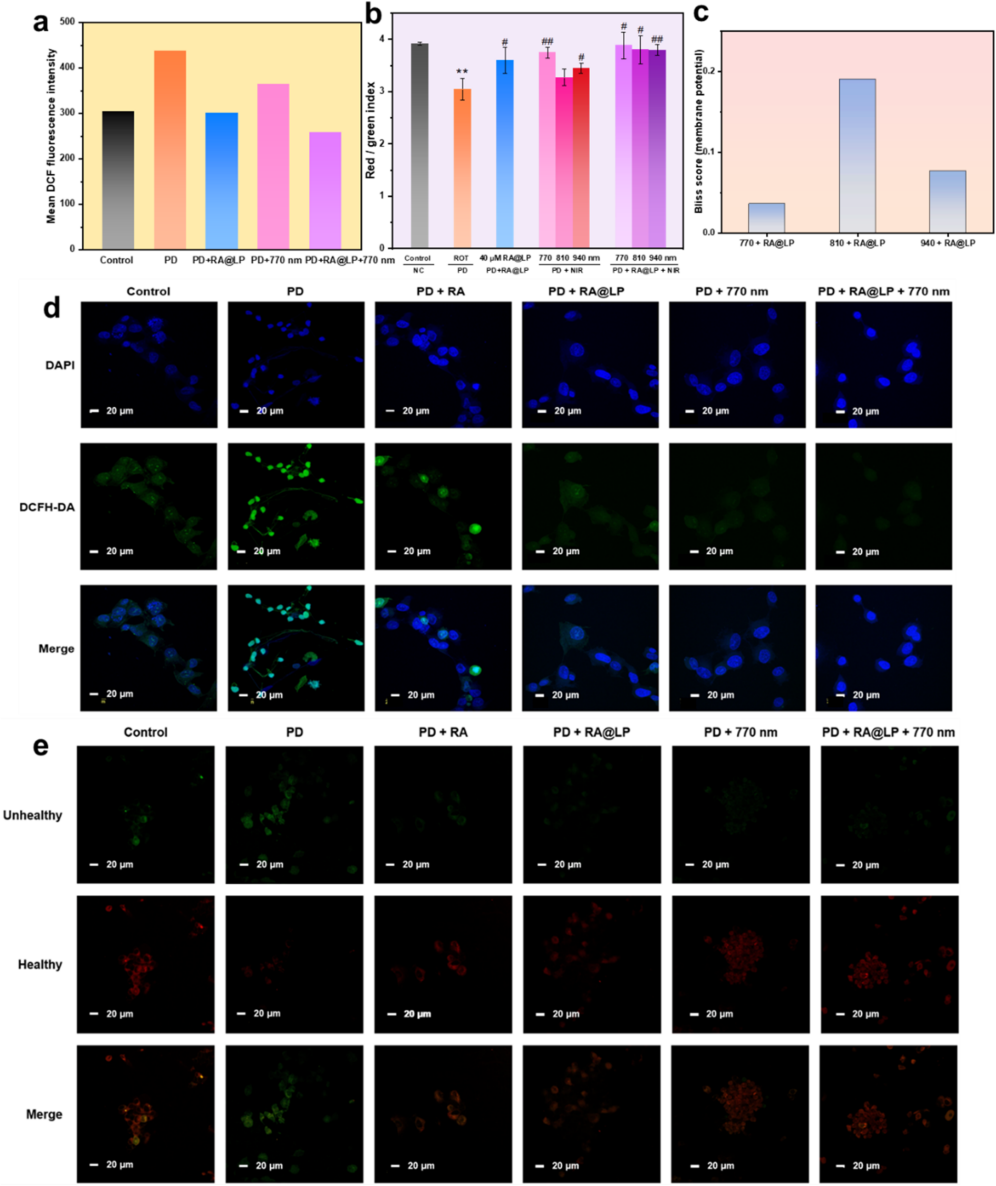
DCF assay exhibited in (a) fluorescence intensity and (d) confocal microscopy. The JC-1 assay is shown in (b) fluorescence intensity and (e) confocal microscopy. Bliss synergy score of (c) membrane potential. The significance is marked as *p < 0.05, **p < 0.01, and ***p < 0.001 compared to the control group. The significance is marked as #*p* < 0.05, ##*p* < 0.01, and ###*p* < 0.001 compared to the ROT group.

In contrast, in the PD group, the proportion of normal cells decreased to 20%, while cells experiencing higher oxidative stress (10^2^ to 10^3^ signal range) increased to 80%. After different treatments, the proportion of normal cells increased in all groups. The combined RA@LP and 770 nm treatment groups showed the most significant improvement, with the normal cell proportion rising to 59%. This suggests that the treatment effectively reduces intracellular ROS levels, lowering oxidative stress and restoring cells to a normal state, thereby demonstrating antioxidant activity. To assess the impact of treatments on cellular mitochondria, cells were stained with JC-1 dye, as shown in Figures 7b,e and S12a. In healthy mitochondria, JC-1 molecules tend to accumulate in mitochondria in aggregated conditions and emit red fluorescence upon excitation.^29^ In contrast, in unhealthy cells, the low potential of mitochondria fails to retain JC-1 molecules in the mitochondria. As a result, JC-1 exists as a monomer and emits green fluorescence. The imaging results demonstrated that the PD group displayed a significant green fluorescence. Post-treatment observations showed a decrease in green fluorescence accompanied by an increase in red fluorescence, with the combined RA@LP and 770 nm treatment groups (PD + RA@LP + 770 nm) exhibiting the most significant increase in red fluorescence. These findings indicate that the treatment method effectively restores mitochondrial function. The synergistic effects of mitochondrial membrane potential changes were magnified in Figure 7c. The combination of three wavelengths all showed a synergistic effect on improving mitochondrial membrane potential, revealing the mitochondrial condition, and boosting efficacy. In our previous studies, near-infrared (NIR) wavelengths were shown to penetrate sufficiently to reach brain tissue and improve outcomes in neurodegenerative disease models.^30^ NIR photobiomodulation primarily exerts its biological effects through interaction with cytochrome c oxidase, which exhibits distinct absorption peaks at approximately 620, 680, 760, and 820 nm. Among the three NIR wavelengths investigated in this study, 770 nm closely corresponds to a functionally relevant absorption band of cytochrome c oxidase, particularly associated with its redox-active centers, thereby more effectively enhancing the enzymatic activity, promoting ATP production, and regulating reactive oxygen species (ROS). The superior performance observed at 770 nm may further arise from its complementary interaction with RA@LP, in which the NIR PBM efficiently activates mitochondria and ATP synthase and modulates ROS generation. At the same time, RA@LP fine-tunes the ROS signaling threshold to sustain efficient ATP synthesis and enhances cellular antioxidant defense, together enabling more effective restoration of redox homeostasis. Conversely, excessive excitation without antioxidant buffering reduces the coupling efficiency, while weaker wavelengths fail to trigger sufficient activation. These outcomes collectively support the concept that PBM and redox modulation synergize through feedback regulation of mitochondrial energy transduction, offering mechanistic insight into how wavelength-specific photobiomodulation can restore bioenergetic homeostasis in PD-related cellular dysfunction.

### Analysis of Cell Apoptosis

To understand the effects of treatment methods on cell apoptosis, cells were stained with FITC-labeled Annexin V and 7-AAD dyes.^31^ During early apoptosis, phosphatidylserine (PS), normally located on the inner leaflet of the cell membrane, flips over to the outer membrane surface. Annexin V binds specifically to PS, and when labeled with FITC, this binding emits green fluorescence, indicating cells in early apoptosis. In late apoptosis, the integrity of the cell membrane is lost, allowing 7-AAD to enter the cell and bind to DNA, producing red fluorescence, which marks cells in the late apoptosis stage. Confocal microscopy images of Annexin V/7-AAD-stained cells are shown in Figure S11. The results indicate that the PD exhibited significant green and red fluorescence signals, indicating high levels of early and late apoptotic cells. In contrast, the treated groups showed reduced green and red fluorescence signals. Among them, the combined treatment group with RA@LP and 770 nm light (PD + RA@LP + 770 nm) showed the weakest green and red fluorescence signals, indicating fewer cells undergoing early and late apoptosis after treatment. This suggests that the combined RA@LP and 770 nm light therapy possesses antiapoptotic effects.

### *In Vivo* Safety Evaluation of NIR Irradiation

To address the reviewer’s concern regarding in vivo safety and tissue tolerance, we performed an in vivo safety evaluation using the same NIR parameters as those applied in vitro (80 mW total power, 30 mW/cm^2^ power density, 15 min). Mice were irradiated with 770 nm NIR light on their dorsal (back) skin for 15 min. In Figure S12b, no visible skin damage, burns, or abnormal tissue responses were observed during or after irradiation, demonstrating that these NIR parameters are well tolerated in vivo and do not cause overt tissue injury.

## Experimental Section

### Synthesis of RA@LP

4.5 mg of DPPC, 0.88 mg of DSPE-PEG2000, and 0.25 mg of DPPA were weighed and mixed and dissolved in 10 mL of ethanol. This lipid solution was added to 0.2 mL of 20 mM RA stock solution in EtOH to prepare RA@LP. After thorough mixing, the solution was transferred to a culture dish and left overnight to allow solvent evaporation, forming a thin lipid film. A surfactant mixture consisting of 0.4 mL of glycerol, 8 mg of Pluronic-F-127, and 3.6 mL of deionized water was then added to the lipid film. This mixture was shaken at room temperature for 2 h to ensure uniform distribution. The lipid solution was then homogenized using an ultrasonic tissue homogenizer for 3 min, with cycles of 10 s of vibration followed by 5 s of rest. Finally, the biologically utilizable size liposomes were acquired through a dialysis process for 2 days.

### 2,2-Diphenyl-1-picrylhydrazyl (DPPH) Assay

The 2,2-diphenyl-1-picrylhydrazyl (DPPH) assay is widely used for evaluating antioxidant activity utilizing a lipophilic free radical reagent. The deep purple DPPH free radical, exhibiting a maximum absorption at 517 nm, turns pale yellow when it is reduced by antioxidants. The antioxidant activity of a sample is assessed by measuring the change in DPPH absorption. The procedure involved dissolving 100 μM of the DPPH reagent and 30 μM of the antioxidant drug in ethanol, and the mixture was stirred in the dark for 1 h. The maximum absorption of DPPH at 517 nm was measured by using UV–vis spectrophotometry. The remaining amount of reactive oxygen species (ROS) was calculated using eq 1, and the drugs’ free radical scavenging activity (RSA) was determined using eq 2.

1 $$\mathrm{ROS}\%=\frac{\mathrm{Abs}\;\mathrm{sample}}{\mathrm{Abs}\;\mathrm{control}}\times 100\%$$

2 $$\mathrm{RSA}\%=\frac{\left(\mathrm{Abs}\;\mathrm{control}-\mathrm{Abs}\;\mathrm{sample}\right)}{\mathrm{Abs}\;c\mathrm{ontro}l}\times 100$$

### Stability Assay of RA@LP

This stability assay evaluated nanoliposome composite materials’ pharmacokinetic properties and circulatory stability. RA@LP of 0.2 mL was dispersed in either 1.8 mL of PBS, deionized water, or serum solution to simulate different physiological conditions, and the mixtures were incubated at 37 °C. Their stability was assessed by monitoring changes in particle size and ζ-potential over six h using a nanoparticle analyzer. For serum stability, freshly prepared LP and RA@LP (1.0 mL) were mixed with an equal volume of FBS. The resulting mixtures were incubated on a horizontal shaker at 70 rpm and 37 ± 1 °C. At predetermined time intervals, 200 μL aliquots were collected, diluted 10-fold with distilled water, and analyzed for particle size for comparison.

### Encapsulation Efficiency of RA@LP

Encapsulation efficiency (EE) is a key indicator for evaluating a nanocarrier’s ability to encapsulate drugs, while loading concentration (LC) represents the actual concentration of drug encapsulated within the material. To assess the effectiveness of nanoliposomes in encapsulating RA, the encapsulation efficiency was determined by the characterization of the absorption peak of RA at 328 nm. The calibration curve of RA absorbance versus concentration was set up in advance. EE and LC were calculated using eqs 3 and 4, respectively.

3 $$EE\left(\%\right)=\frac{Abs\;of\;sample-Abs\;of\;\mathrm{LP}}{Abs\;of\;excepted}\times 100\%$$

4 LC(μM)=encapsulationconcentration×EE

### SH-SY5Y Cell Line Cultivation

In this study, the SH-SY5Y human neuroblastoma cell line was deployed for cellular investigation. SH-SY5Y cells were cultured in DMEM/F12 supplemented with 10% FBS and 1% P/S. The cells are maintained at 37 °C in a humidified incubator with 5% CO_2_, with confluency around 70–80%. The medium was changed every 2 days.

### SH-SY5Y Cell Line Differentiation

To differentiate SH-SY5Y cells into an appropriate neuronal phenotype for Parkinson’s disease research, this study employed a 2-staged approach using controlled culture media. The base culture medium consisted of EMEM/F-12, 10% FBS, and 1% P/S. The differentiation process was carried out in two stages. In the first stage, 0.01 mM retinoic acid was added to the culture medium as a differentiation agent. For the second stage, the aforementioned retinoic acid and 0.05 μg/mL brain-derived neurotrophic factor (BDNF) were introduced into the culture medium.

### Hemolysis Assay

Fresh blood collected from mice was centrifuged at 1000–1500*g* for 5–10 min at 4 °C to pellet red blood cells. A hemolysis assay was performed by measuring the absorbance at 540 nm. The positive control consisted of completely lysed blood cells, while the negative control contained only mouse blood in PBS. Supernatants from blood samples incubated with **LP** and **RA@LP** were measured at 540 nm to determine the extent of RBC lysis. The hemolysis rate was calculated by using eq 5.

5 $$\%\mathrm{hemolysis}=\frac{A_{s}-A_{0}}{A_{\infty }-A_{0}}$$

where *A*_s_ is the absorbance of the samples at 540 nm, *A*_0_ is the absorbance of the negative control at 540 nm, and *A*_∞_ is the absorbance of the positive control at 540 nm.

### BBB Model

A Transwell coculture system with 0.4 μm pore size was used to establish an in vitro BBB model. SH-SY5Y cells were seeded in the lower chamber at 10^5^/well and cultured for 24 h. BMECs were then seeded on the apical surface of inserts at 5 × 10^4^/well and cultured until confluent. The inserts were placed into wells containing SH-SY5Y cells, and coculture was maintained for 1 day to allow barrier maturation. The samples were added to the upper chamber for 8 h for the investigation of the BBB penetration ability.

### Rotenone-induced PD cellular model

This study utilized rotenone (ROT), an inhibitor of mitochondrial complex I, to establish the cellular model of PD. The rotenone was dissolved in dimethyl sulfoxide (DMSO), with a final concentration of DMSO at 0.01%. In brief, differentiated cells were seeded in 96-well plates at 10^5^ cells/mL concentrations and left for 24 h. After a pioneer assay for optimizing ROT concentration, culture media containing 50 μM ROT were used to induce damage to the cells, and the cells were incubated for another 24 h.

### NIR LED Treatment of PD Cells

The 770, 810, and 940 nm NIR LED panels were supplied by Everlight Co. Ltd. in this study. Each LED generates a total power output of 80 mW and a power density of 30 mW/cm^2^, which CAS 140CT Compact Array Spectrometer calibrated. The LED chips were strategically arranged in a two-dimensional configuration with a size of 27 mm × 36 mm, which is strategically designed to fit the arrangement of a segment of a 96-well plate. Each NIR LED treatment session lasted for 15 min, followed by incubation for 24 h. The cells were then subjected to further cellular analysis.

### RA@LP Treatment of PD Cells

The treatment of RA@LP on PD cells was carried out by dissolving RA@LP in the medium with various final concentrations (20, 40, 60, 80, and 100 μM). RA@LP was incubated with the cells and was removed after 24 h for further cellular analysis.

### NIR LED Complexed with RA@LP Treatment of PD Cells

NIR LED complexed with RA@LP treatment on PD cells was carried out by initially dissolving RA@LP in the medium with various final concentrations (20, 40, 60, 80, and 100 μM). RA@LP was incubated with the cells and was removed after 24 h for further cellular analysis.

### Cell Viability Test

After the treatment, 10% Alamar blue solution was added to the wells and incubated for 1 h. Using a multimode microplate reader, the cell viability was then determined by measuring fluorescence at 590 nm with an excitation wavelength of 530 nm.

### Superoxide Dismutase (SOD) Activity Analysis

Superoxide dismutase (SOD) is an enzyme that converts superoxide radicals into oxygen and hydrogen peroxide through dismutation, protecting cells from oxidative damage, and is a crucial antioxidant enzyme for resisting oxidative stress. To understand the antioxidant capacity of different treatment methods, SOD activity analysis was used to evaluate the cellular antioxidant ability. WST-1 method, which was first uncovered by Ukeda et al.,^32^ exerts WST-1 to investigate the activity of SOD. WST, a tetrazolium reagent, as an indicator of superoxide, can be reduced by superoxide to form a water-soluble formazan compound with an absorption peak at 438 nm. At the same time, SOD negatively impacts the concentration of superoxide, reducing the rate of WST-1 reduction. For SOD activity analysis, an SOD assay kit (Abcam, ab65354, USA) was applied in this research. A standard curve of WST inhibition versus SOD activity was established in advance. After the treatment, cells were lysed with cell lysis buffer at 4 °C for 20 min and centrifuged at 14,000*g* for 3 min, and the supernatant was retained to measure absorbance at 438 nm using a multimode microplate reader.

### JC-1 Assay

To understand the degree of mitochondrial recovery in treated SH-SY5Y cells, a JC-1 assay was conducted. JC-1, developed by Smiley et al.,^33^ is a cationic lipophilic dye that mitochondria can absorb. JC-1 aggregates as polymers and exhibits red fluorescence in well-conditioned mitochondria, which possess a normal membrane potential. In contrast, in apoptotic cells with low mitochondrial membrane potential, their mitochondria cannot absorb large amounts of JC-1, and JC-1 exists as monomers and exhibits green fluorescence. Thus, JC-1 can assess mitochondrial conditions by fluorescence detection. The procedure follows the protocol provided by the supplier (Abcam, ab112134, USA). After the treatment of cells, diluted JC-1 was added and incubated for 30 min. Afterward, buffer B was added, and fluorescence was measured using a multimode absorbance and fluorescence spectrophotometer. Green fluorescence, peaked at 525 nm, excited by 490 nm, and red fluorescence, peaked at 590 nm, excited at 540 nm, were measured for estimation.

### Western Blot Assay

Cellular lysates were prepared using RIPA buffer and clarified by centrifugation at 14,000 rpm. Equal amounts of protein (30 μg) were separated on 15% SDS-PAGE gels and transferred to PVDF membranes. After being blocked with 5% BSA, membranes were incubated overnight at 4 °C with ATP5C1 antibody (1:1000), followed by HRP-conjugated secondary antibody (1:10000). Protein bands were visualized using ECL Plus detection. GAPDH was used as a loading control, and band intensities were quantified with ImageJ.

### ATP Detection Assay

Cell lysates were prepared by rinsing the cells with prechilled PBS, lysing them with ice-cold 1× ATP detection sample buffer, and homogenizing the mixture by pipetting. Plasma and serum samples were centrifuged and filtered through 10 kDa spin filters before dilution with a 1× ATP Detection Sample Buffer. The assay reaction mixture, containing 1× ATP detection assay buffer, ATP detection D-Luciferin, and ATP detection luciferase, was added to standard wells (10 μL of ATP standard, with a range of 12 fmol to 10 pmol), blank wells (10 μL of sample buffer), and sample wells (10 μL sample). Plates were incubated at room temperature in the dark for 15–20 min, then luminescence was measured within 5 min. ATP concentrations were calculated from a log-transformed standard curve.

### Bliss Synergy Score Analysis

The Bliss score, as a synergy index, was calculated to assess the interactive effect between the RA@LP treatment and PBM treatments on the cell viability, ATP5C1 expression, SOD activity, ATP production, and mitochondrial membrane potential. The effect of each treatment was determined using eq 6.

6 $$E_{\mathrm{treatment}}=\frac{X_{\mathrm{treatment}-}X_{\mathrm{control}}}{X_{\mathrm{control}}-X_{\mathrm{ROT}}}$$

where *E*_treatment_ stands for the effect of a certain treatment, *X*_treatment_ stands for the experimental data of the treatment, *X*_control_ stands for the experimental data of the control group, *X*_ROT_ stands for the experimental data of the control group, where *E*_NIR_ and *E*_RA@LP_ represent the individual production of the PBM treatment and the RA@LP treatment, respectively. A Bliss synergy score greater than 0 indicates a synergistic interaction; a Bliss synergy score of 0 denotes an additive effect; and a Bliss synergy score less than 0 suggests an antagonistic relationship between the treatments. The score was determined using eq 7.

7 $$\mathrm{bliss}\;\mathrm{synergy}\;\mathrm{score}=\frac{E_{\mathrm{NIR}+\mathrm{RA}@\mathrm{LP}}}{E_{\mathrm{NIR}}+E_{\mathrm{RA}@\mathrm{LP}}-E_{\mathrm{NIR}}\times E_{\mathrm{RA}@\mathrm{LP}}}$$

### *In Vivo* NIR Irradiation Safety Evaluation

All animal experiments were conducted in accordance with National Yang Ming Chiao Tung University institutional guidelines and approved protocols (IACUC no. 1141205). For the *in vivo* safety assessment, mice were anesthetized and the dorsal (back) skin was exposed to NIR irradiation using a 770 nm light. The irradiation parameters were identical to those used in the in vitro experiments, with a total power of 80 mW, a power density of 30 mW/cm^2^, and an irradiation duration of 15 min. After treatment, the irradiated skin area was carefully examined for visible signs of damage, including erythema, burns, or ulceration. Mice were further monitored for abnormal tissue responses following irradiation.

## Conclusion

Despite centuries of scientific endeavor and remarkable medical advancements, PD remains one of medicine’s most formidable challenges, standing as an enigmatic fortress that continues to resist our therapeutic arsenal. The quest for a definitive treatment has proven to be an intricate labyrinth, where conventional approaches, such as levodopa, provide symptomatic relief but have yet to diminish all of the disorder’s symptoms and side effects. Mitochondria, as the power plant of the cells, are profoundly connected to the function of dopaminergic neurons. Mitochondrial dysfunction creates a devastating cascade of cellular problems. Antioxidants and NIR, bearing the merits of cleaving ROS and elevating mitochondrial function, are potential approaches for the therapy of PD. These findings represent a significant advance in PD treatment strategies, moving beyond traditional symptom management to address fundamental cellular mechanisms of neurodegeneration. The dual-modal approach offers a promising therapeutic strategy by combining stable drug delivery with targeted PBM therapy, potentially providing a more comprehensive treatment option for PD patients.

## Supplementary Material



## Notes

NIR PBM corresponds to the absorption spectrum of mitochondria and the mechanism of action of RA to remove ROS in cells (Figure S1); synthesis flowchart of nanoliposomes complexed with RA (Figure S2); the size distribution (Figure S3a,b) and the serum stability (Figure S3c) of LP and RA@LP; the spectral graph of liposomes containing different concentrations of RA (Figure S4); representative photographs of Transwell system at different stages of the blood–brain barrier model development (Figure S5); optical microscopy images of SH-SY5Y cells at different days of differentiation (Figure S6); flowchart of PD cell model establishment and treatment procedure (Figure S7); the Western blot gels of ATP synthase (Figure S8a) and GADPH (Figure S8b) Flow cytometric analysis of DCFH-DA fluorescence intensity (Figure S9); bar chart showing the proportion of cells before and after the 10^2^ DCF fluorescence intensity threshold (Figure S10); confocal microscopy images of cells from different treatment groups stained with Annexin V/7-AAD (Figure S11); JC-1 assay of mitochondrial membrane potential in cells treated with LP alone (Figure S12a) and *in vivo* safety assessment of 770 nm NIR irradiation on the mouse dorsal skin for 15 min (Figure S12b) (PDF)
