# Supplementary material for: Synergistic Neuroprotection in Parkinson’s Disease via Photobiomodulation and Liposomal Rosmarinic Acid Delivery
Source: ACS Biomater Sci Eng. 2026 Jan 26;12(2):906–21. doi: 10.1021/acsbiomaterials.5c01969 (PMC12892251; doi:10.1021/acsbiomaterials.5c01969)
Supplement: Supplementary file 1 [file ab5c01969_si_001.pdf]

Supporting Information

Synergistic Neuroprotection in Parkinson's Disease via Photobiomodulation and Liposomal Rosmarinic Acid Delivery

Ting-Yi Su,[a] *Chen-Ya Wang,*[a] *Wen-Tse Huang,*[a] *Ming-Yang Chang,*[a] *Ming-Hsien Chan,*[b,*] *and Ru-Shi Liu*[a,*]

[a] Department of Chemistry, National Taiwan University, Taipei 106, Taiwan.

[b] Department of Biomedical Imaging and Radiological Sciences, National Yang Ming Chiao Tung University, Taipei 112, Taiwan.

Corresponding Authors

*Ming-Hsien Chan (mhchan@nycu.edu.tw)

*Ru-Shi Liu (rsliu@ntu.edu.tw)

Supplementary figures

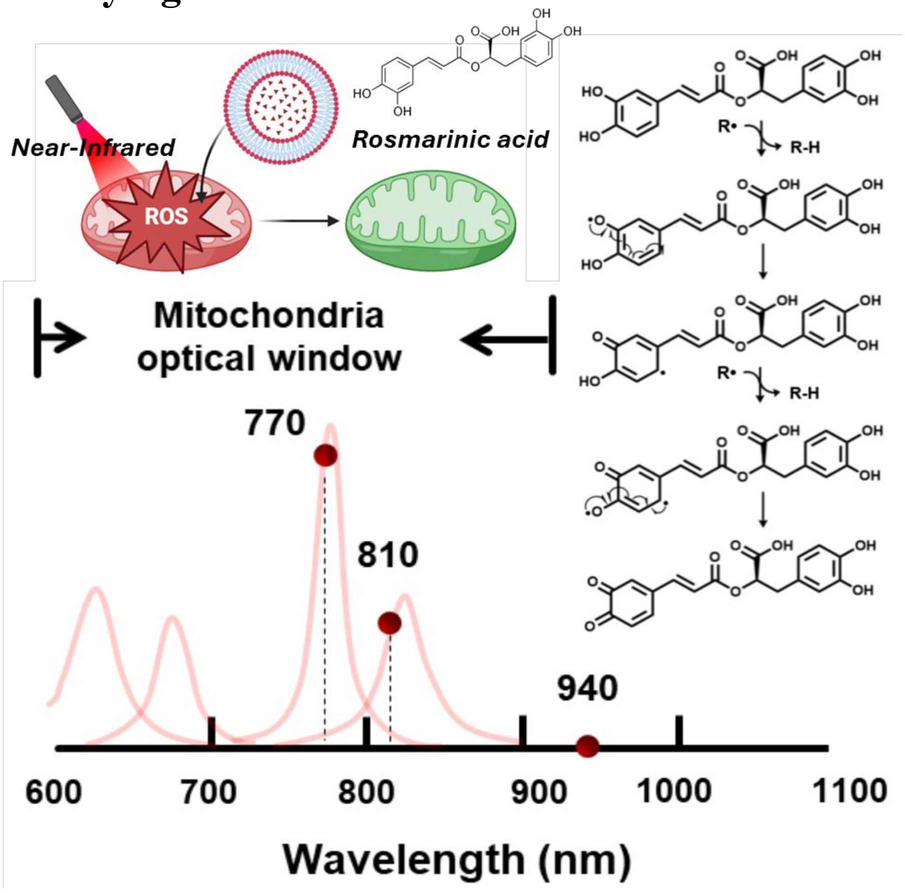

Figure S1. NIR PBM corresponds to the absorption spectrum of mitochondria and activates the energy cycle and the mechanism of action of RA to remove ROS in cells.

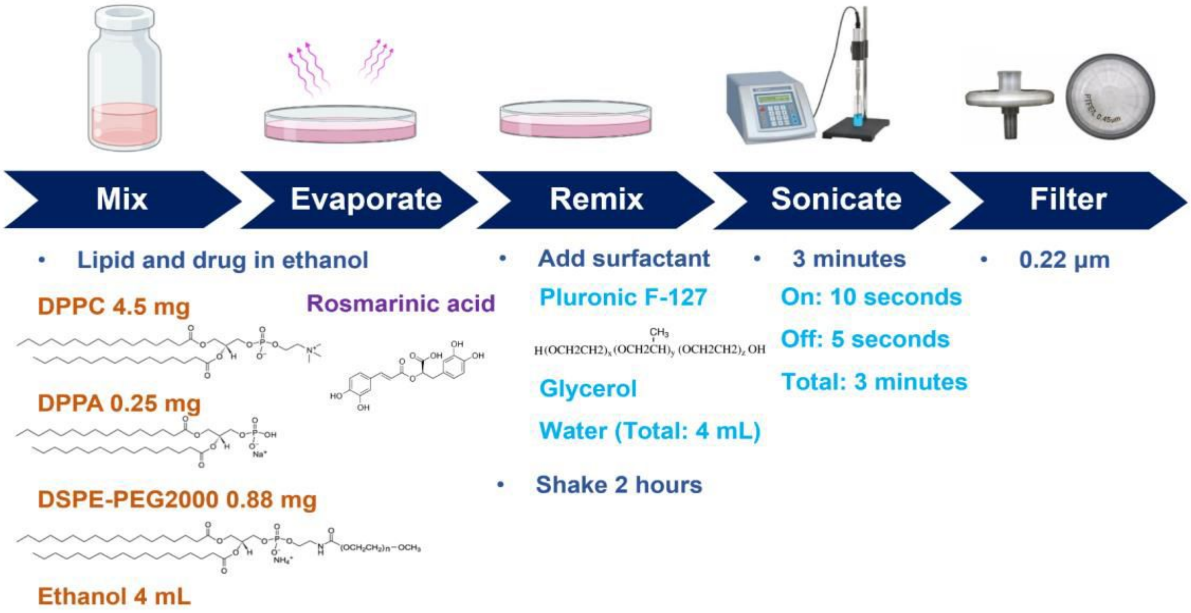

Figure S2. Synthesis flow chart of nanoliposomes complexed with RA.

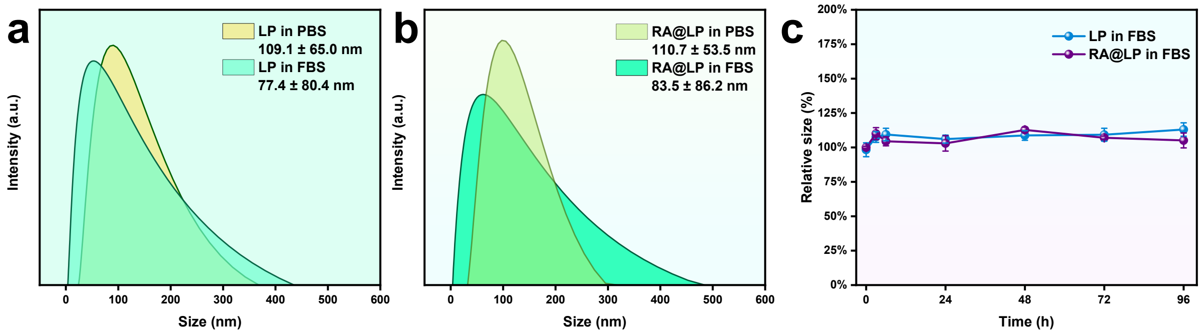

Figure S3 Size distribution of (a) LP and (b)RA@LP in the PBS and FBS environment. (c) Serum stability test of LP and RA@LP over 96 hours.

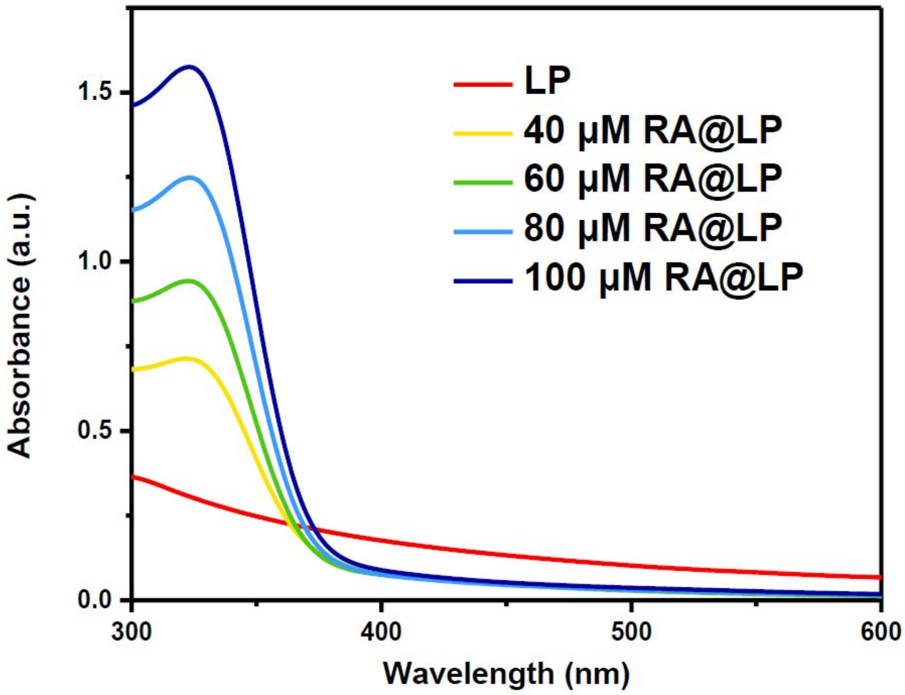

Figure S4. The spectral graph (300–600 nm) of liposomes containing different concentrations of RA.

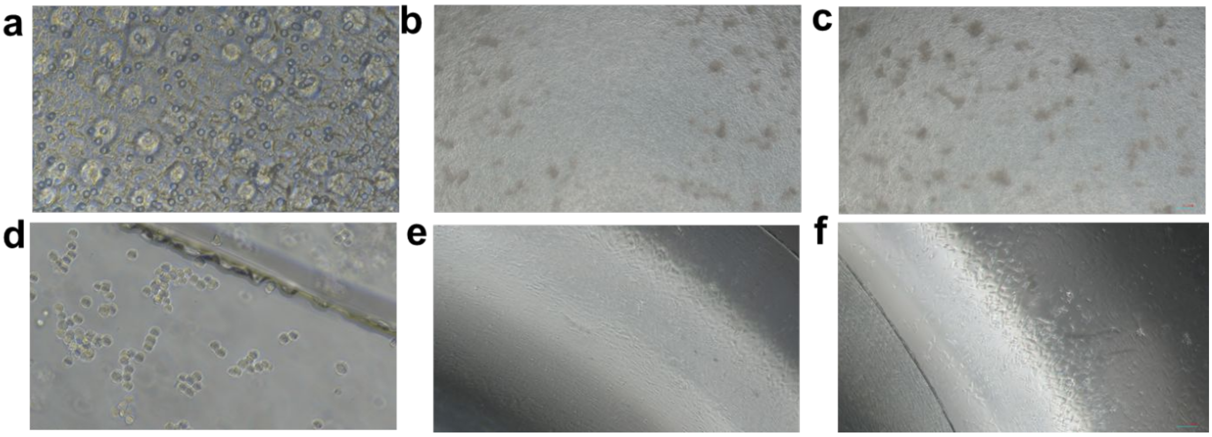

Figure S5. Representative photographs of transwells at different stages of the blood-brain barrier model development. Initial cell seeding on the (a) apical and (d) brain sides. Confluent monolayers were achieved on the (b) apical and (e) brain sides. Transwells following 8-hour exposure to RA@LP on the (c) apical and (f) brain sides.

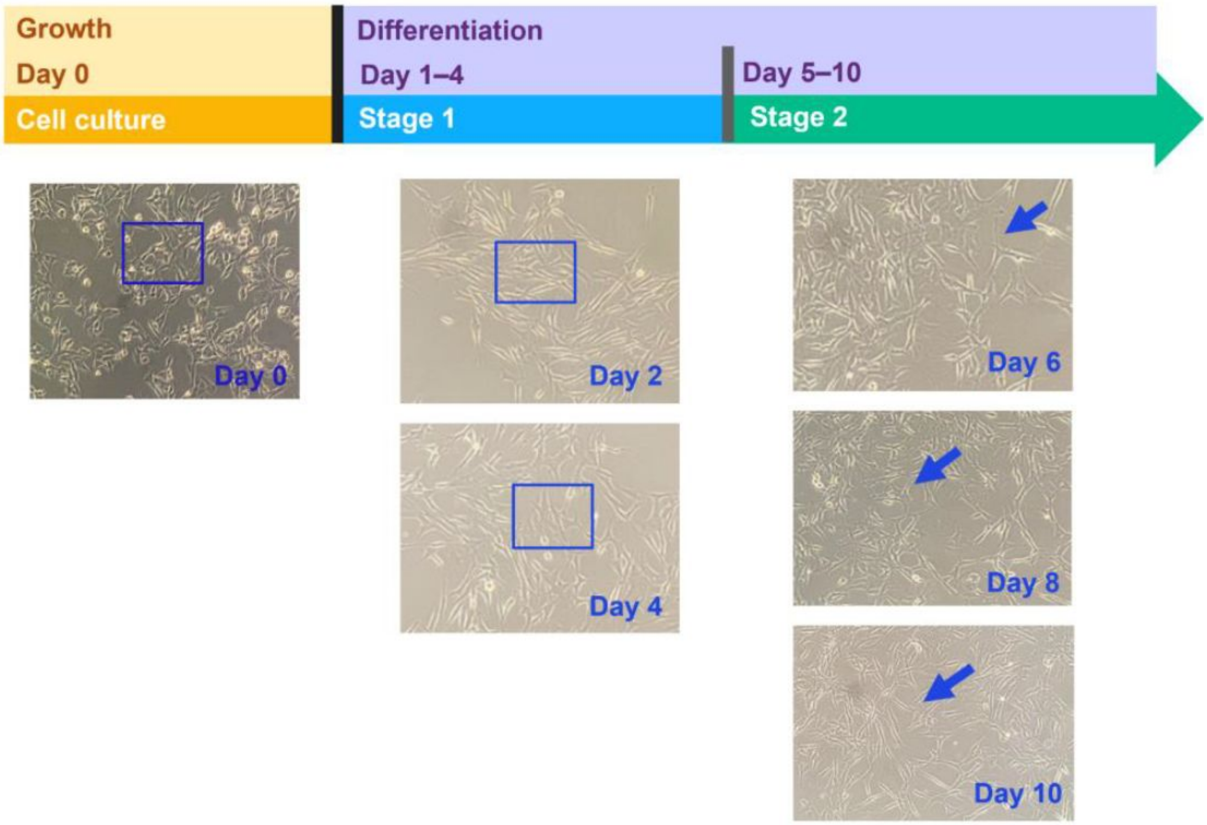

Figure S6. Optical microscopy images of SH-SY5Y cells at different days of differentiation.

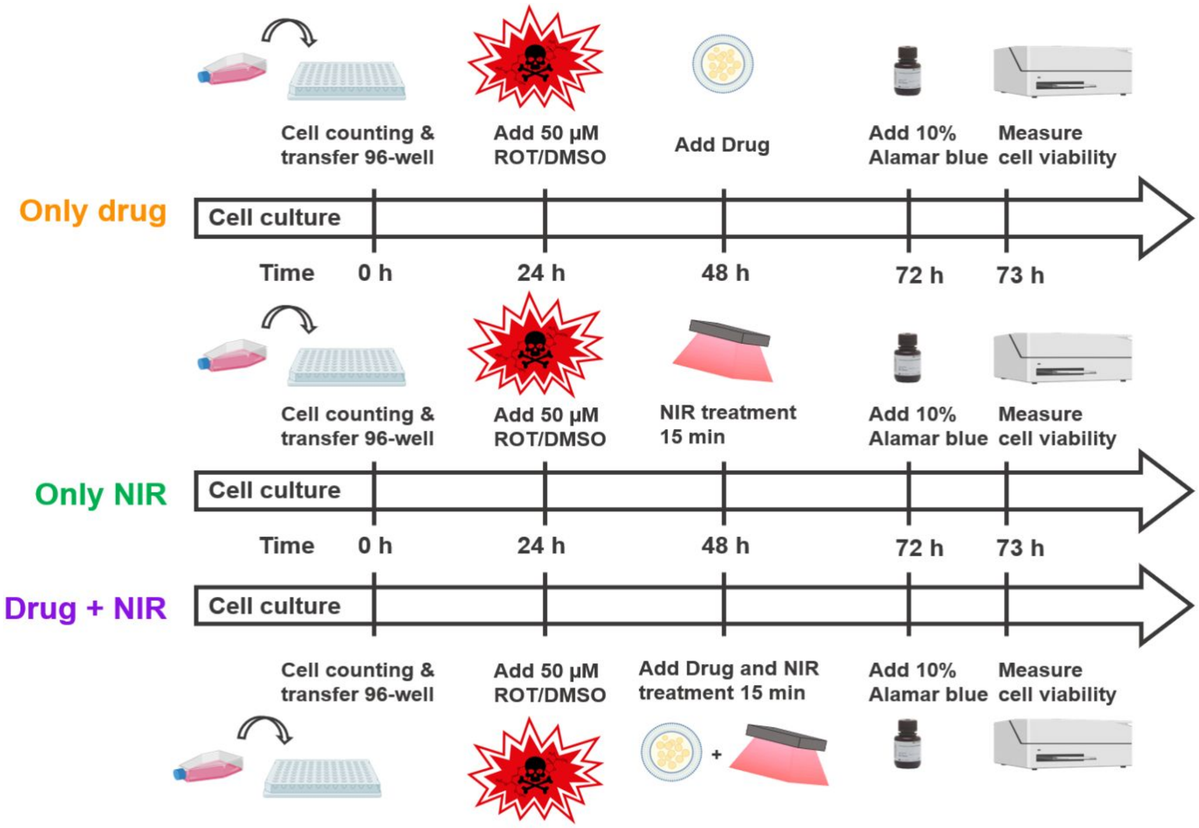

Figure S7. Flowchart of PD cell model establishment and treatment procedure.

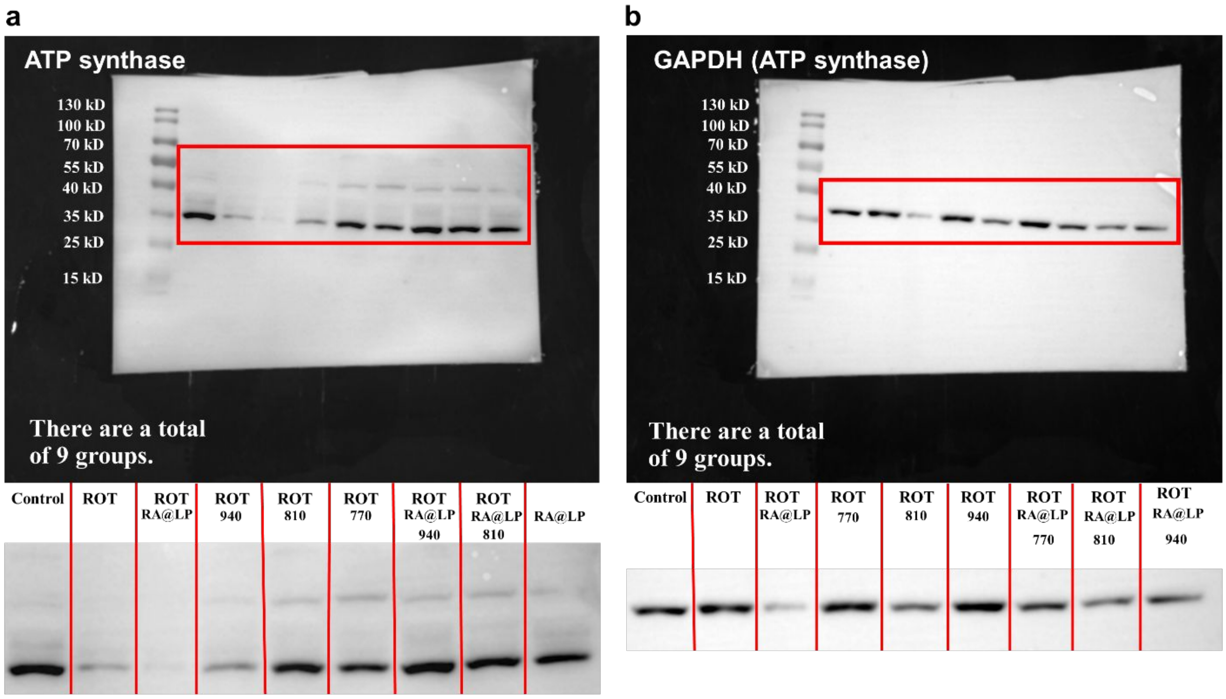

Figure S8. The western blot gels of (a) ATP synthase and (b) GADPH.

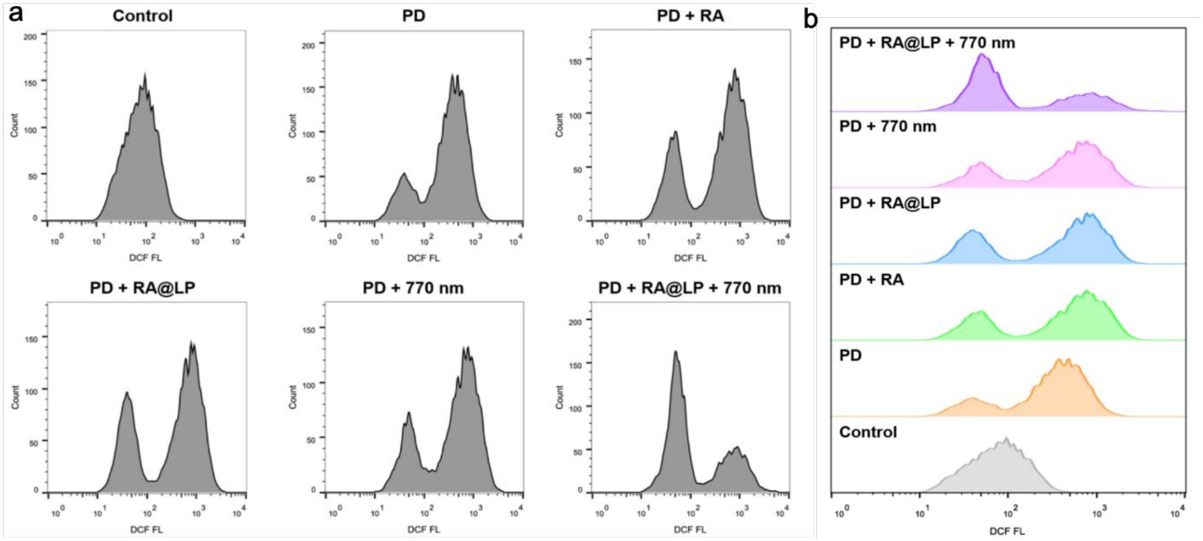

Figure S9. Flow cytometric analysis of DCFH-DA fluorescence intensity. (a) Flow cytometry results for each treatment group. (b) Overlay plot of flow cytometry results from all treatment groups.

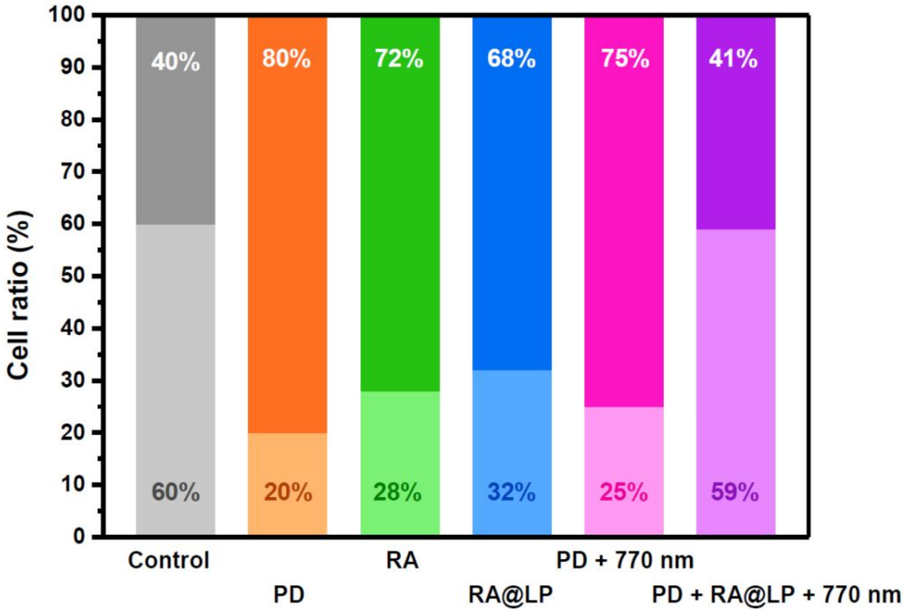

Figure S10. Bar chart showing the proportion of cells before and after the 10^2 DCF fluorescence intensity threshold.

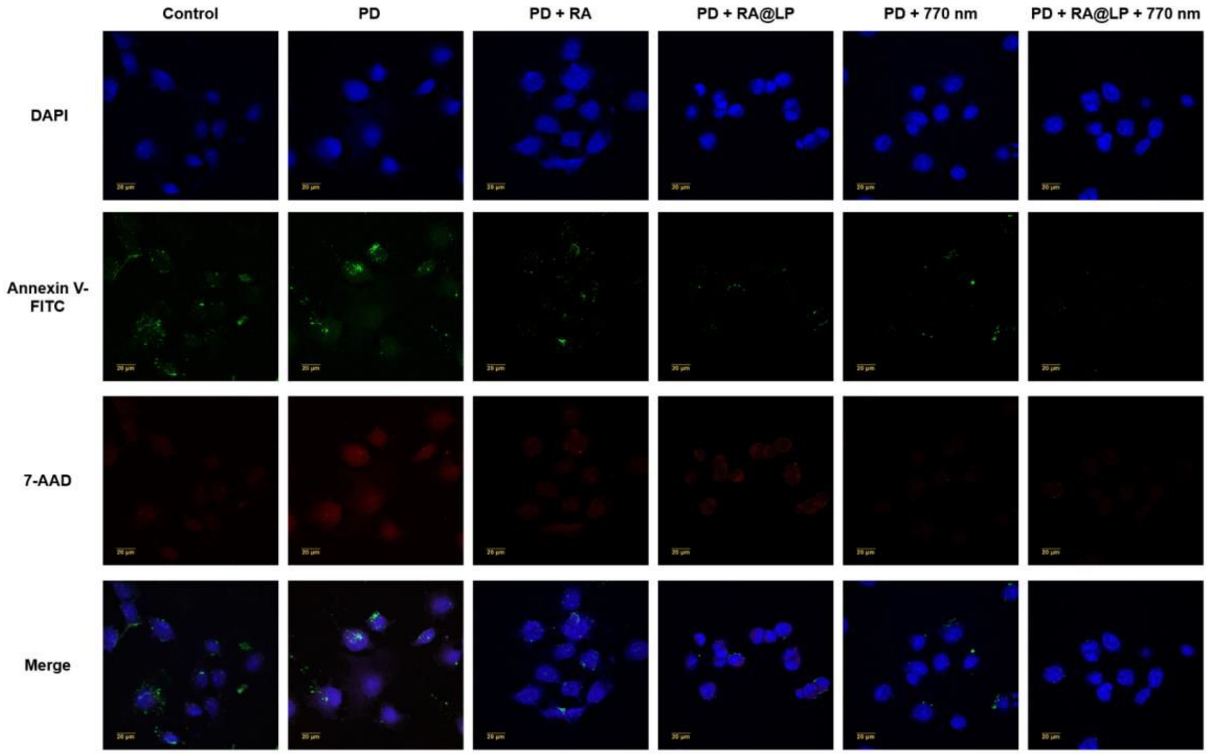

Figure S11. Confocal microscopy images of cells from different treatment groups stained with Annexin V/7-AAD.

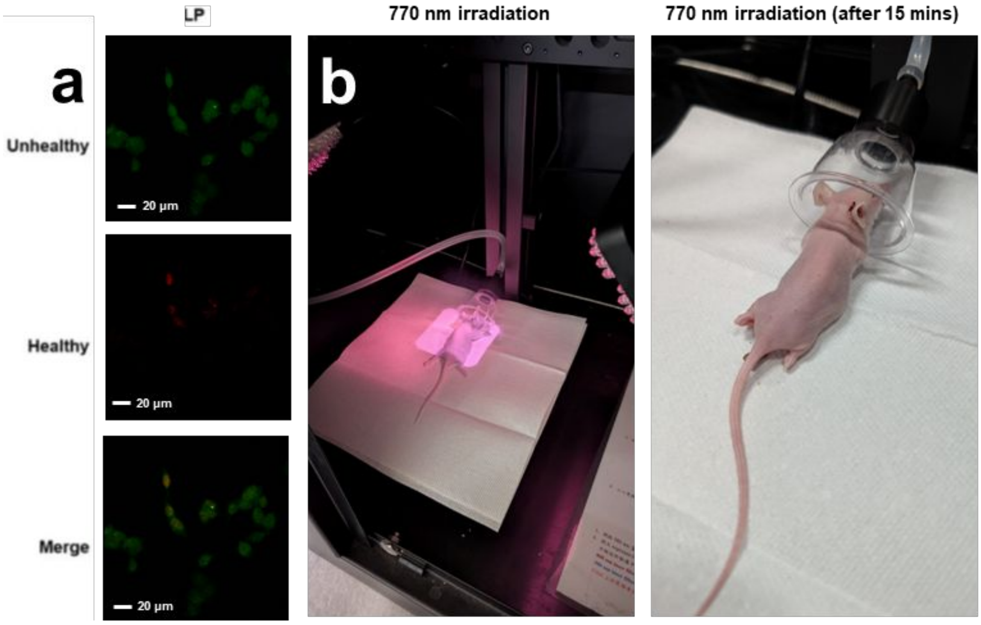

Figure S12. (a) JC-1 assay of mitochondrial membrane potential in cells treated with LP alone. (b) *In vivo* safety assessment of 770 nm NIR irradiation on the mouse dorsal skin for 15 min.

Supplementary Tables

Table S1. Regression equations and % inhibition values for SOD standards at different concentrations.

SOD Standard Concentration	Regression Curve Equation	% Inhibition
Blank (0 U/mL)	y = 0.0556x + 0.661	-
0.625 U/mL	y = 0.0370x + 0.426	33.5%
1.25 U/mL	y = 0.0355x + 0.444	36.3%
2.5 U/mL	y = 0.0217x + 0.384	61.0%
5 U/mL	y = 0.0173x + 0.273	68.9%
10 U/mL	y = 0.0104x + 0.216	81.3%
20 U/mL	y = 0.0113x + 0.264	79.7%

Table S2. Regression equations and % inhibition values for different treatment groups.

Treatment groups	Regression Curve Equation	% Inhibition
Blank	y = 0.0556x + 0.661	-
NC	y = 0.0463x + 0.315	16.7%
PD	y = 0.0612x + 0.283	-10.1%
PD + RA	y = 0.0584x + 0.393	-5.04%
PD + RA@LP	y = 0.0571x + 0.427	-2.70%
PD + 770 nm	y = 0.0585x + 0.462	-5.22%
PD + 810 nm	y = 0.0602x + 0.442	-8.27%
PD + 940 nm	y = 0.0597x + 0.433	-7.37%
PD + RA@LP + 770 nm	y = 0.0530x + 0.482	4.68%
PD + RA@LP + 810 nm	y = 0.0558x + 0.443	-0.360%
PD + RA@LP + 940 nm	y =0.0553x + 0.454	0.540%
